# Green synthesis of polypyrrole-SnO_2_ nanocomposites using *Foeniculum vulgare* extract for crystal violet adsorption and solvent-dependent radical scavenging

**DOI:** 10.1039/d5ra09693f

**Published:** 2026-02-03

**Authors:** Priya Kaushik, Ruchi Bharti, Renu Sharma, Annu Pandey

**Affiliations:** a Department of Chemistry, University Institute of Sciences, Chandigarh University Punjab India ruchi.uis@cumail.in; b Department of Fibre and Polymer Technology, KTH Royal Institute of Technology Stockholm Sweden annua@kth.se

## Abstract

The development of multifunctional nanomaterials provides new opportunities to address both environmental and biomedical challenges. In this study, SnO_2_ nanoparticles were synthesized using *Foeniculum vulgare* seed extract and subsequently incorporated into independently synthesized polypyrrole (APS-mediated oxidative polymerization) to obtain PPy-SnO_2_ nanocomposites. Comprehensive structural, optical and morphological analyses, including FTIR, UV-Vis spectrophotometry, XRD, SEM-EDS, HRTEM, DLS, zeta potential, and BET, confirmed the successful formation of the nanocomposites and the uniform incorporation of SnO_2_ within the PPy matrix. The PPy-SnO_2_ nanocomposites demonstrated significant adsorption performance for crystal violet, achieving 92% removal under optimized conditions, including pH 7, a dye concentration of 10 ppm, 50 mg adsorbent, and 50 °C for 150 min. Adsorption behaviour followed a pseudo-2nd-order kinetic model, and a maximum capacity of 162.6 mg g^−1^ estimated from the Langmuir isotherm was achieved. The antioxidant activity assessed by DPPH and ABTS assays in methanol and hexane showed higher radical scavenging efficiency in methanol, achieving 90.8% inhibition at 800 µg mL^−1^. PPy-SnO_2_ consistently outperformed pure polypyrrole, indicating the significant role of SnO_2_ in enhancing electron-transfer-based scavenging. Overall, these results highlight the PPy-SnO_2_ NCs as an effective dual-application material that combine strong antioxidant properties with high-efficiency dye removal to provide a sustainable approach for environmental remediation.

## Abstract 2

Green-synthesized multifunctional nanomaterials were developed with enhanced adsorption efficiency and antioxidant activity, demonstrating their potential for sustainable environmental applications.

## Graphical abstract

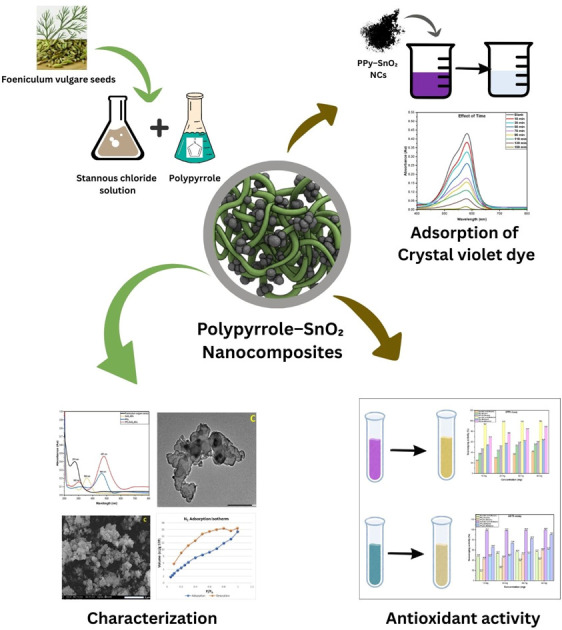

## 1. Introduction

Water contamination by synthetic dyes remains a significant environmental challenge due to their high chemical stability, complex aromatic structures and resistance to biodegradation.^1–3^ These dyes are widely used across various industries, including textiles, pharmaceuticals, and chemicals, resulting in substantial volumes of wastewater containing these pollutants. The discharge of dye-polluted effluents into water bodies not only degrades water quality but also reduces light penetration, disrupting aquatic ecosystems and harming photosynthetic organisms. Moreover, many synthetic dyes are toxic and carcinogenic, posing serious risks to both aquatic organisms and human health.^4,5^ Conventional treatment methods, such as coagulation, particle aggregation, biological treatment and activated carbon adsorption, are typically insufficient for complete dye removal, highlighting the development of more efficient and environmentally friendly remediation strategies.^6–8^ Alongside water contamination, oxidative stress induced by reactive oxygen species (ROS) is a primary biological and industrial concern. ROS such as superoxide anions, hydroxyl radicals, and hydrogen peroxide can cause lipid peroxidation, DNA damage, protein denaturation, and accelerated material degradation.^9,10^ Antioxidants play a pivotal role in neutralizing ROS and preventing oxidative damage.^11,12^ Beyond their biological significance, antioxidants are widely employed in industrial and material applications, including food preservation, cosmetics, and protection of polymers or metals.^13,14^ Consequently, the development of multifunctional materials capable of both dye removal and antioxidant activity offers a promising dual-benefit approach.

Conducting polymers such as polypyrrole (PPy) have drawn considerable interest due to their unique chemical, physical and electrical properties.^15,16^ PPy is known for its high chemical and thermal stability, good biocompatibility, large surface area and tunable conductivity, making it suitable for adsorption, catalysis and electrochemical applications.^17,18^ When combined with metal nanoparticles, such as tin (Sn), the resulting nanocomposites can further enhance the properties of the material.^19,20^ Metal nanoparticles increase surface area, enhance electron transfer, and provide additional functional sites while contributing to free radical scavenging.^21^ The synergistic interaction between PPy and SnO_2_ enables the development of dual-functional nanocomposites that can simultaneously perform pollutant removal and antioxidant activity. Previous studies have demonstrated that PPy-based nanocomposites are effective for removing cationic dyes such as crystal violet (CV). For example, PPy-chitosan and PPy-bentonite composites have shown high dye adsorption capacities under various conditions.,^22,23^ while PPy-metal oxide and PPy-metal nanocomposites have been examined for free radical scavenging activity.^24,25^ However, reports on plant-assisted Polypyrrole tin oxide nanocomposites (PPy-SnO_2_ NCs) with dual functionality—combining high-capacity dye adsorption and concentration-dependent antioxidant activity across multiple solvents—are limited.^26^ In particular, *Foeniculum vulgare*-assisted synthesis has not been explored in depth, despite the fact that the extract effectively facilitates green nanoparticle formation. This comparison highlights the gap in the literature that our study employs *Foeniculum vulgare* seed extract only for the green synthesis of SnO_2_ nanoparticles, while polypyrrole is synthesized by oxidative polymerization; then SnO_2_ nanoparticles are incorporated into the PPy matrix to synthesize PPy-SnO_2_ nanocomposites. Green synthesis using eco-friendly reducing agents, such as plant extracts, provides a sustainable alternative to conventional chemical methods.^27,28^ They minimize hazardous residues, enhance biocompatibility, and often result in nanocomposites with improved structural and functional properties.^29^ This study reports a low-toxicity and plant-mediated route for the synthesis of PPy-SnO_2_ NCs and systematically evaluates their dual-functional properties. The synthesized nanocomposites were characterized using UV-vis spectroscopy, FTIR, XRD, SEM, TEM, EDS and DLS to understand their structural, morphological and thermal features.^30–34^ Dye removal efficiency was investigated under varying conditions of NC dose, pH, dye concentration, contact time, and temperature. At the same time, the concentration-dependent antioxidant activity was evaluated in different solvents using standard assays.^35–39^ By elucidating structure–property relationships, this study highlights the potential of PPy-SnO_2_ NCs as sustainable, multifunctional materials for addressing both environmental pollution and oxidative stress.^40,41^ The findings demonstrate potential *in vitro* (chemical) antioxidant and ROS-scavenging activity of PPy-SnO_2_ nanocomposites, suggesting their suitability for future biological studies. This study aligns with the Sustainable Development Goals by facilitating efficient water decontamination (SDG 6), promoting eco-friendly synthesis (SDG 12), and exhibiting antioxidant potential (SDG 3).

## 2. Experimental methodology

### 2.1. Materials

All chemicals and solvents, including pyrrole monomer, ammonium persulfate (APS), stannous chloride (dihydrate), crystal violet dye, 0.1 M HCl, and 0.1 M NaOH, were obtained from Sigma-Aldrich, and distilled water was employed for solution preparation. *Foeniculum vulgare* was sourced from local store in Chandigarh. Equipment included a magnetic stirrer, centrifuge, pH meter, 1000 µL micropipette, cuvettes and a UV-vis spectrophotometer for absorbance measurements.

### 2.2. Preparation of *Foeniculum vulgare* extract

Fresh *Foeniculum vulgare* seeds (20 g) were rigorously rinsed with distilled water to eliminate impurities and immersed in around 400 mL of distilled water. The solution was maintained at 100 °C until the colour of the solution was changed, and the volume was reduced to half. After reaching room temperature, the extract was filtered through Whatman filter paper to yield a pale-yellow solution, which act a natural reducing and capping agents for the green synthesis of tin oxide NPs, ensuring uniform and eco-friendly nanoparticle formation.

### 2.3. Synthesis of polypyrrole

Polypyrrole was synthesized *via* oxidative polymerization by slowly mixing 50 ml of 0.1 M ammonium persulfate (APS, oxidant) solution to 50 ml of 0.1 M pyrrole solution, which was maintained under constant magnetic stirring for 5–6 hours at 10–20 °C. The black precipitate formed was then separated through filtration and rinsed with distilled water. The mixture was further centrifuged and then oven-dried at 70 °C for 6 h.

### 2.4. Synthesis of tin oxide (SnO_2_) nanoparticles

To synthesize SnO_2_ nanoparticles, a solution of 0.1 M stannous chloride dihydrate (50 mL) was prepared and added dropwise to 50 ml of *Foeniculum vulgare* extract with constant stirring. After 30–35 minutes of stirring, a colour changes from colourless to milky yellow was observed that suggested the fabrication of SnO_2_ nanoparticles. The resulting product was centrifuged (3000 rpm, 10 min), rinsed with water and ethanol and dried at 70 °C for 6 h.

### 2.5. Fabrication of polypyrrole-based tin oxide-nanocomposites

To prepare the PPy-SnO_2_ NCs, the SnO_2_ NPs solution was added gradually to the PPy solution for 40–45 minutes with stirring. A 1 : 1 ratio of PPy to SnO_2_ was stirred constantly at room temperature for 24 h to enable complete incorporation of SnO_2_ into the polypyrrole. After 24 h, nanocomposites were centrifuged at 2000 rpm for 15 minutes and washed 2–3 times with distilled water and ethanol, ensuring the removal of unbound organic phytochemicals. Nanocomposites were precipitated, and the remaining filtrate was separated. The precipitate was oven dried at 60 °C for 6 h until dryness was attained. The synthesized nanocomposites were stored in a desiccator for further characterisation and used to examine its efficiency in removing crystal dye from aqueous solution.

## 3. Characterization studies

The synthesized PPy-SnO_2_ NCs were characterized by various analytical techniques. Fourier transform infrared (FTIR) spectra were conducted on a PerkinElmer FTIR spectrometer, and UV-vis absorption spectra were monitored using a Shimadzu UV-1900 spectrophotometer. The morphological features and elemental analysis were accessed *via* scanning electron microscopy with energy-dispersive spectroscopy (SEM-EDS, SU8010 series, HITACHI). Particle size and zeta potential were analyzed with a Zetasizer Nano (Malvern Panalytical, UK) provided with dynamic light scattering, and TEM-EDS was performed using a CRYO ARM™ 300 II instrument.

### 3.1. UV-visible spectral analysis

**Fig. 1:**
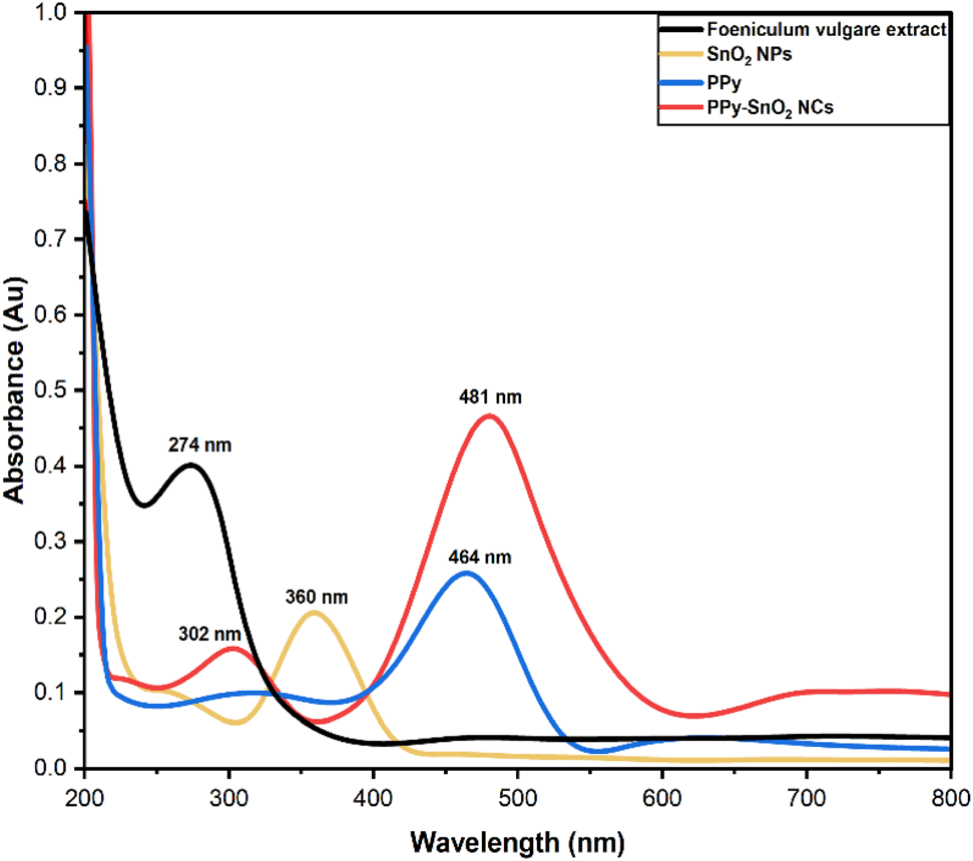
UV-visible spectrum of *Foeniculum vulgare* extract, polypyrrole (PPy), SnO_2_ nanoparticles, and polypyrrole-SnO_2_ NCs.

UV-vis spectroscopy is a commonly employed technique for confirming nanoparticles synthesis and studying their optical characteristics in a liquid medium. In this study, UV-vis spectra of *Foeniculum vulgare* extract, SnO_2_ NPs, polypyrrole (PPy), and PPy-SnO_2_ NCs were recorded with a wavelength range from 200 to 800 nm, as shown in Fig. 1.

The extract exhibited a prominent absorption peak at 274 nm, attributed mainly to π → π* transitions of phytochemicals such as flavonoids and phenolics. These compounds act as natural reducing and stabilizing agents. After the synthesis of SnO_2_ NPs, the spectrum exhibited a new peak at approximately 360 nm, corresponding to the surface plasmon resonance of SnO_2_ or partially oxidized Sn species. The shift in the absorption band from 274 nm to 360 nm and the decreased intensity indicate that the phytochemicals acted as reducing and stabilizing agents during the nanoparticle formation.

PPy exhibited a characteristic absorption band at 464 nm, attributed to π–π* transitions within its conjugated polymer backbone. In the PPy-SnO_2_ NCs, the absorption band was broader and slightly shifted, suggesting strong electronic interactions between PPy chains and SnO_2_ nanoparticles. This spectral change confirms the successful incorporation of SnO_2_ NPs into the PPy matrix, thereby enhancing charge transfer and improving the nanocomposite's stability.^42^

### 3.2. FTIR spectral analysis

**Fig. 2:**
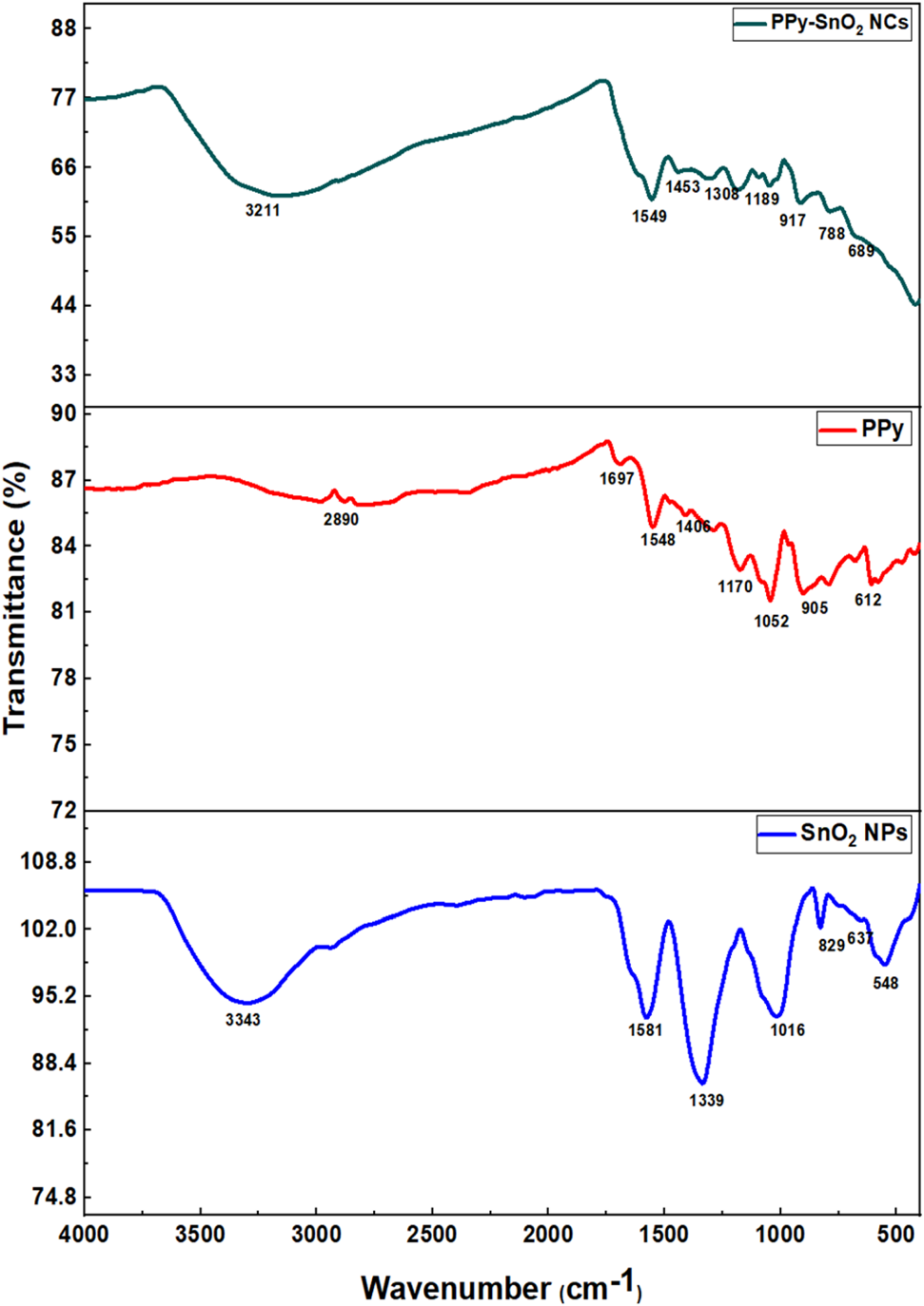
FTIR analysis of PPy, SnO_2_ NPs^N^, and PPy-SnO_2_ nanocomposites.

FTIR spectroscopy of *Foeniculum vulgare* extract, SnO_2_ NPs, PPy, and PPy-SnO_2_ NCs was carried out using the PerkinElmer Fourier-Transform Infrared Radiation spectrophotometer, with a range between 4000 cm^−1^ and 400 cm^−1^ to investigate the chemical bonds and optical performance and study the chemical bonds present in the nanoparticles and nanocomposites (Fig. 2).

The FTIR spectrum of SnO_2_ nanoparticles (SnO_2_ NPs) shows a broad band at 3390–3400 cm^−1^ (O–H stretching) and a peak at ∼1630 cm^−1^ (O–H bending). The strong absorptions in the lower wavenumber region (600–500 cm^−1^) are attributed to Sn–O and Sn–O–Sn vibrations, confirming the presence of Sn–O bonds on the nanoparticle surface.^43^ The FTIR spectrum of pure polypyrrole (PPy) exhibits distinct vibrational bands characteristic of its polymeric structure. Peaks observed at around 1550 and 1460 cm^−1^ are attributed to the C=C and C–C stretching vibrations within the pyrrole ring. The band near 1300 cm^−1^ corresponds to C–N stretching, reflecting the polymer backbone, while the absorption around 1150 cm^−1^ is assigned to in-plane C–H deformation of the pyrrole units. These features confirm the successful formation of the PPy polymer network. The FTIR spectrum of PPy-SnO_2_ NCs exhibits the characteristic PPy peaks at 3400, 1550, 1460, 1300, and 1150 cm^−1^, corresponding to N–H, C=C, C–C, C–N, and in-plane C–H vibrations, respectively. Minor shifts and variations in peak positions are observed, indicating interactions between the PPy matrix and the SnO_2_ nanoparticles. Additionally, a distinct absorption peak in the 600–500 cm^−1^ region, assigned to Sn–O and Sn–O–Sn vibrations, confirms the successful incorporation of SnO_2_ and the formation of stable PPy-SnO_2_ NCs.^44^

### 3.3. X-ray diffraction analysis

**Fig. 3:**
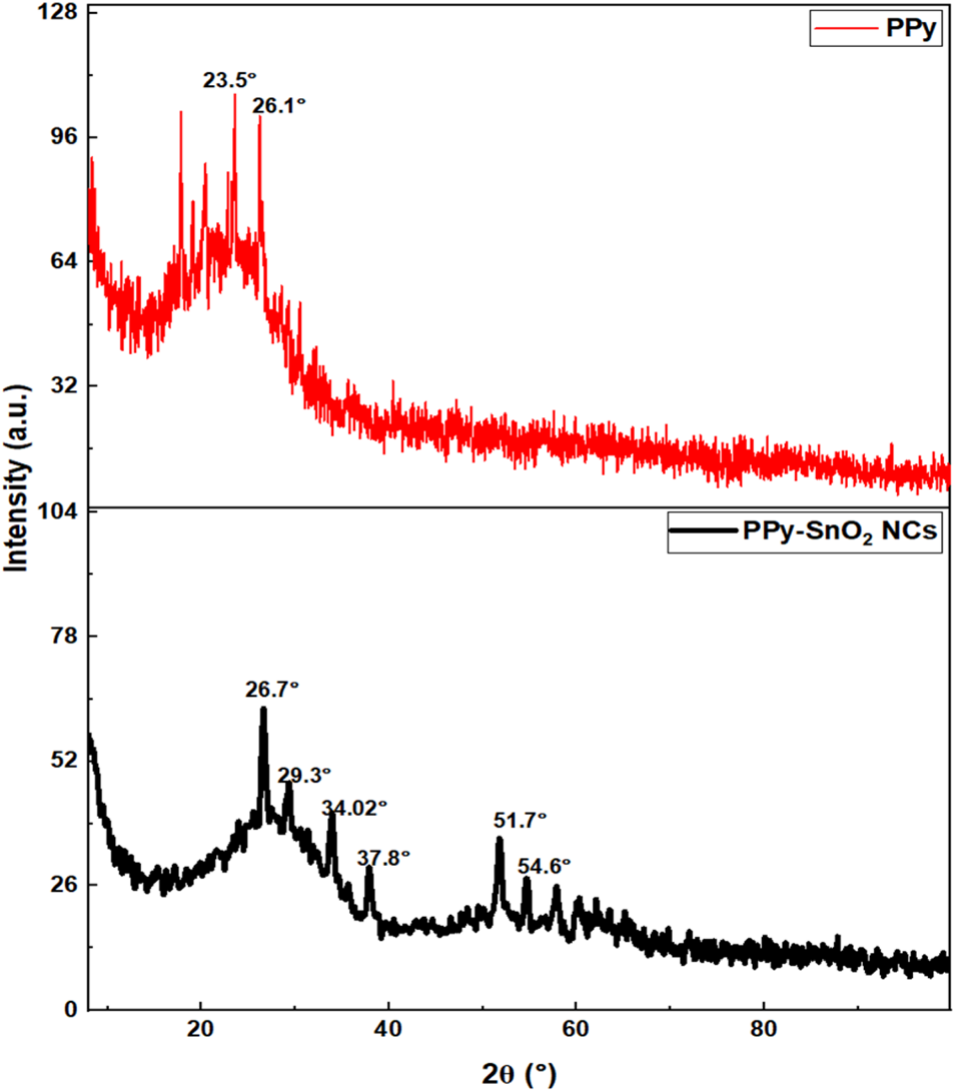
XRD analysis of synthesized PPy and PPy-SnO_2_ NCs.

The XRD analysis was performed to examine the structural properties of PPy and PPy-SnO_2_ NCs, as illustrated in Fig. 3. The diffraction pattern of pure PPy demonstrated two distinct broad peaks at 2*θ* ≈ 23.5° and 26.1°, which is characteristic of the predominantly amorphous nature of conducting polymers. PPy exhibited a crystallinity of 42.6% with the remaining 57.4% attributed to the amorphous phase. The average crystallite size of PPy was calculated to be approximately 16.3 nm, consistent with the range reported in previous studies of PPy-based nanostructures.

The average crystallite size was estimated using the Debye–Scherrer equation,
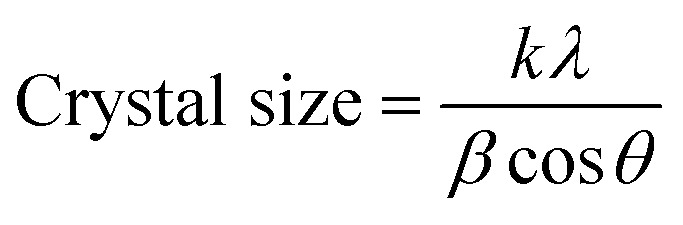
where *D* is the crystal size, *K* is the shape factor (0.94), *λ* is the wavelength oIf incident Cu Kα X-ray radiation (0.1542 nm), *β* is the full width at half maximum of the diffraction peak (FWHM), *θ* is the Bragg's angle, also expressed in radians.

The PPy-SnO_2_ nanocomposites demonstrated sharp diffraction peaks over the amorphous polymer background, ensuring the presence of crystalline SnO_2_ NPs within the PPy matrix. Various sharp peaks observed at 2*θ* = 26.7°, 34.02°, 37.8°, 51.7° and 54.6° corresponds to (110), (101), (200), (211), and (220) planes of rutile SnO_2_, respectively (JCPDS No. 41-1445). This suggests the strong interactions among SnO_2_ NPs and PPy. The crystallinity of the nanocomposites improved moderately to 51.9% with less amorphous content (48.1%). Crystallite size was calculated to be 28.8 nm using the Debye–Scherrer equation, confirming the successful incorporation of SnO_2_ into the PPy scaffold and the development of a nanocomposite structure, consistent with earlier reports of enhanced crystallinity in PPy-based nanocomposites upon incorporation of a metal.^45^

### 3.4. Scanning-electron microscopy with energy dispersive X-ray spectroscopy (SEM-EDS)

**Fig. 4:**
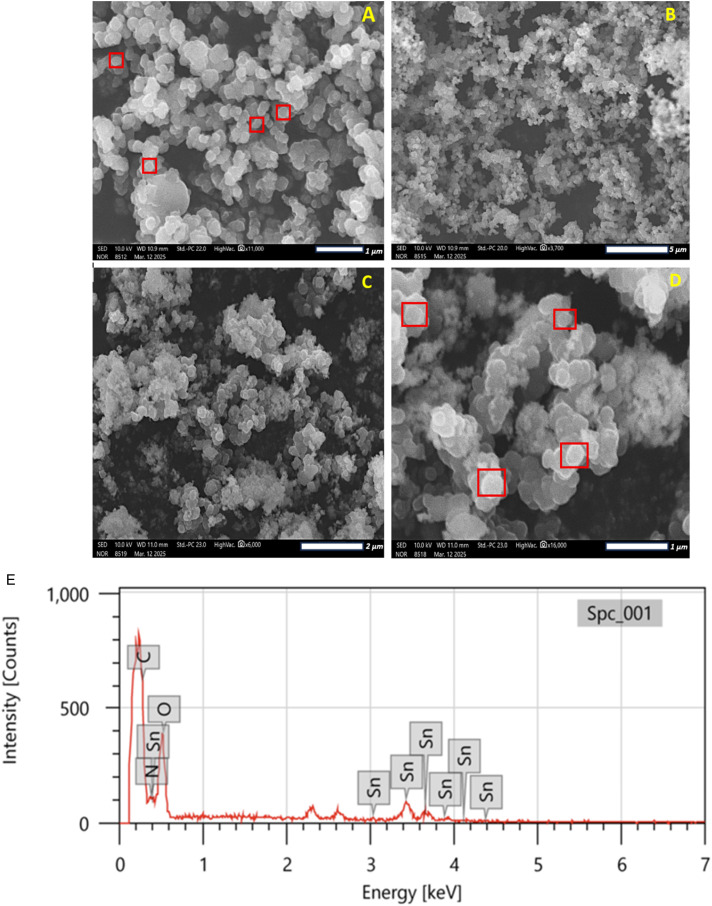
SEM image of (A and B) PPy and (C and D) PPy-SnO_2_ NCs at different magnifications (E) SEM-EDS of synthesized PPy-SnO_2_ nanocomposites.

The surface morphology and elemental analysis of PPy and PPy-SnO_2_ NCs were accessed by SEM-EDS on a HITACHI SU8010 series microscope. SEM enables high-resolution images of the sample through scanning with a focused electron beam, enabling detailed information about particle morphology, shape, and surface features. As shown in Fig. 4a and b, PPy particles exhibited a rough, irregular and porous surface, while SnO_2_ NPs mainly appeared spherical in shape with some irregularity. The PPy-SnO_2_ nanocomposites displayed irregularly aggregated, spherical particles with relatively smooth domains, as illustrated in Fig. 4c and d. These morphological features are consistent with those observed in prior studies of PPy-SnO_2_ composites.^46^

The average particle size of the PPy-SnO_2_ nanocomposites, calculated using ImageJ, was approximately 48 nm, which aligns well with previously reported values for similar polymer-metal nanocomposites. The EDS spectra, as shown in Fig. 4e, exhibited clear peaks for tin (Sn), carbon (C), nitrogen (N), and oxygen (O), validating the successful embedding of SnO_2_ within the PPy framework. Sn was observed as the predominant element, while C, N, and O corresponded to the characteristic composition of the PPy matrix. These results indicate that the synthesized PPy-SnO_2_ nanocomposites exhibit a uniform, well-defined morphology. The quantitative elemental analysis is presented in Table 1. Overall, the SEM and EDS analyses confirm the successful integration of SnO_2_ nanoparticles into the PPy matrix, forming a stable dual-component nanocomposite. The apparent difference between mass% and atom% is expected in EDS quantification. In particular, Sn shows a higher mass% but lower atom % because Sn has a much higher atomic weight than C, N and O; therefore, fewer Sn atoms contribute disproportionately to the total mass percentage.

**Table 1:** EDS analysis of the PPy-SnO_2_ nanocomposites

Element	Line	Mass%	Atom%
C	K	14.1 ± 0.18	26.6 ± 0.34
N	K	3.8 ± 0.30	5.4 ± 0.41
O	K	36.3 ± 0.83	57.8 ± 1.17
Sn	L	45.8 ± 1.44	10.2. ± 0.27
Total		100.00	100.00

### 3.5. High-resolution transmission electron microscopy

**Fig. 5:**
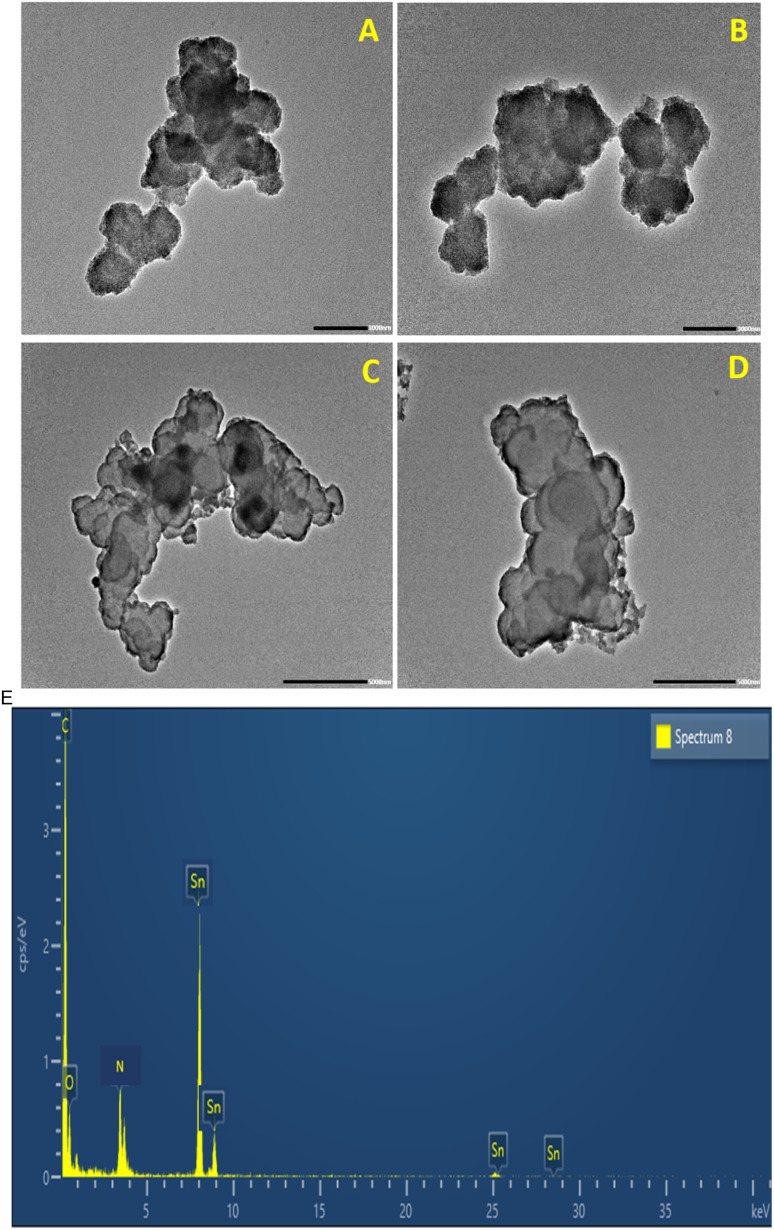
HRTEM image of synthesized (A and B) PPy and (C and D) PPy-SnO_2_ NCs (E) HRTEM-EDS analysis of PPy-SnO_2_ Nanocomposites.

The high-resolution transmission electron microscopy (HRTEM) images of PPy and PPy-SnO_2_ nanocomposites revealed distinct morphological features. The HRTEM micrographs of PPy (Fig. 5) revealed aggregation of irregularly shaped nanoparticles with an amorphous character, consistent with its broad diffraction features observed in XRD [46]. In the PPy-SnO_2_ NCs, as illustrated in Fig. 5, SnO_2_ nanoparticles are incorporated in the polymer matrix and exhibit irregular and aggregated clusters. The lighter-coloured areas show the PPy matrix, whereas the darker areas illustrate the high-electron-density SnO_2_ nanoparticles.

The crystalline nature of the SnO_2_ nanoparticles is revealed by well-resolved lattice planes with an interplanar spacing of approximately 0.27 nm, which correlates to the (101) plane of tetragonal SnO_2_ (JCPDS No. 04-0673), consistent with similar PPy-metal systems.^47^ Furthermore, the HRTEM images of PPy-SnO_2_ NCs further show that SnO_2_ nanoparticles are predominantly spherical and uniformly distributed within the PPy matrix, with individual particle sizes (10–15 nm) and aggregate dimensions (50–150 nm) determined directly from the micrographs using ImageJ software. Energy dispersive X-ray spectroscopy (EDS) analysis confirms the presence of Sn along with N, O and C, corresponding to the composition of the PPy-SnO_2_ NCs as mentioned in Table 2. As shown in Table 2, the higher Sn mass% relative to atom% is consistent with EDS reporting due to higher atomic weight of Sn compared to the lighter elements (C, N and O).

**Table 2:** HRTEM analysis of the PPy-SnO_2_ nanocomposites

Element	Line	Mass%	Atom%
C	K	13.5 ± 0.2	44.9 ± 0.3
N	K	2.5 ± 0.19	6.8 ± 0.2
O	K	29.5 ± 0.8	29.1 ± 0.9
Sn	K	54.5 ± 1.2	19.2 ± 0.3
Total		100	100

### 3.6. Dynamic light scattering analysis (DLS)

**Fig. 6:**
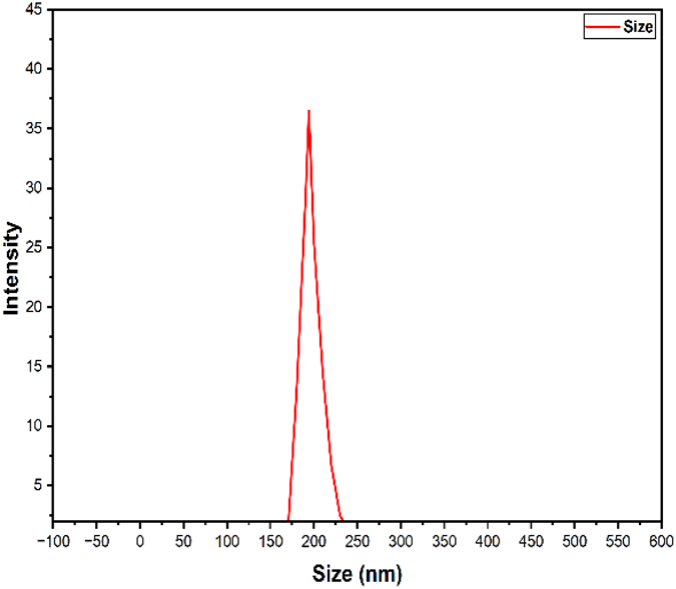
Hydrodynamic size distribution of PPY-SnO_2_ nanocomposites in aqueous suspension obtained by dynamic light scattering (DLS).

Dynamic Light Scattering (DLS) analysis was performed on the synthesized PPy-SnO_2_ nanocomposites in aqueous suspension to determine the hydrodynamic particle size distribution. The nanocomposites had an average hydrodynamic diameter (Z-average) of 194.1 nm, as shown in Fig. 6, which is considerably larger than the crystallite size determined by XRD (28 nm) and the particle sizes observed in SEM. This difference is attributed to the presence of solvation layers and particle aggregation in aqueous media, which are commonly observed in polymer-based nanocomposites. The corresponding polydispersity index (PDI) of 0.212 indicates a moderately narrow size distribution, reflecting good uniformity and dispersion of the nanocomposites. Such behaviour is well documented in the literature, especially for polymer-stabilized and green-synthesized nanomaterials, where biological moieties and interparticle interactions can increase the hydrodynamic size. Comparable DLS values for PPy-based nanocomposites have been reported in the range of 120–183 nm, which align with the present observation.^48^ The single peaked DLS distribution further confirms the formation of a relatively homogeneous population of PPy-SnO_2_ nanocomposites in aqueous dispersion. This hydrodynamic behaviour is particularly relevant for aqueous adsorption systems, where dispersion stability governs effective surface availability.

### 3.7. Zeta-potential

**Fig. 7:**
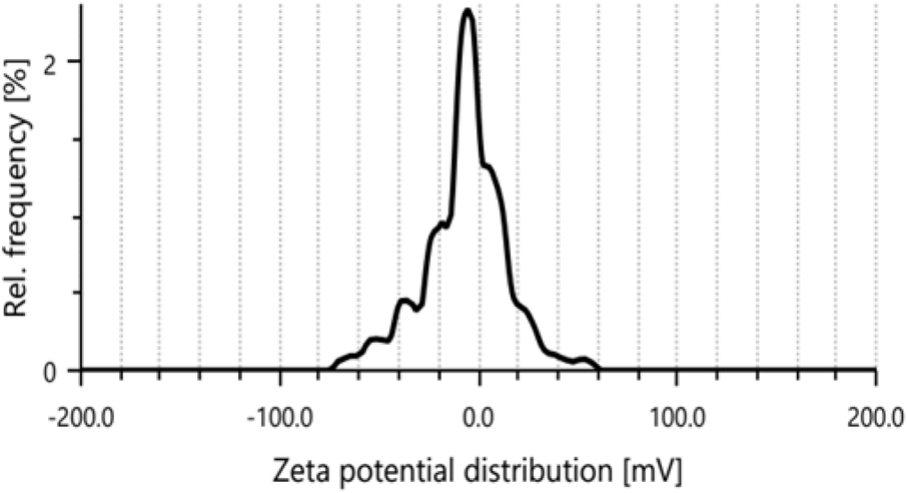
Zeta potential of PPy-SnO_2_ nanocomposites, indicating surface charge and colloidal stability.

The zeta potential of the synthesized PPy-SnO_2_ NCs was assessed to evaluate their colloidal stability. The nanocomposites exhibited a mean zeta potential value of −10.2 mV (Fig. 7), indicating a moderate negative surface charge that provides sufficient electrostatic repulsion to prevent aggregation.^49^

This suggests that the synthesized nanocomposites are colloidally stable and well-dispersed in aqueous medium. The distribution peak at −4.6 mV, along with a standard deviation of 0.6 mV, indicates a relatively uniform particle dispersion in the aqueous medium.

Additional parameters, including a mean intensity of 710.1 kcounts s^−1^, filter optical density of 2.8738, conductivity of 0.475 mS cm^−1^, electrophoretic mobility of 0.7947 µm cm V^−1^ s^−1^, and transmittance of 17.7%, corroborate the colloidal stability of the PPy-SnO_2_ NCs. These findings confirm that the synthesized nanocomposites are well-dispersed and exhibit robust stability in aqueous suspension, which is critical for their potential applications.^50^

### 3.8. BET-analysis

**Fig. 8:**
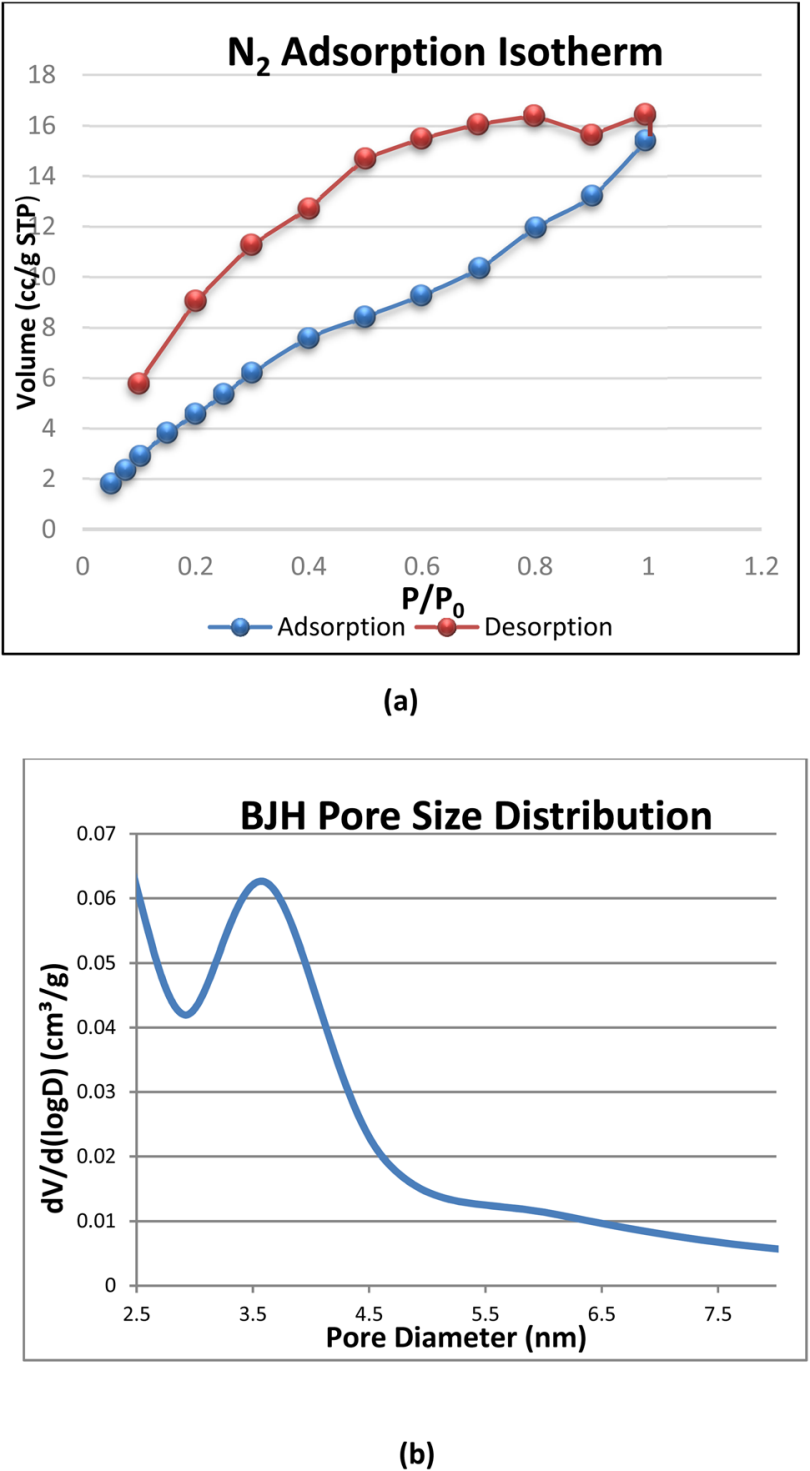
(a) Nitrogen adsorption–desorption isotherm of PPy-SnO_2_ NCs exhibiting type IV behaviour, confirming mesoporosity, (b) exhibiting a BET surface area of 24.14 m^2^ g^−1^.

The surface properties of synthesized PPy-SnO_2_ NCs were analyzed with nitrogen adsorption–desorption isotherms at 77 K, and the data were reported by applying the Brunauer–Emmett–Teller (BET), *t*-plot (DeBoer), and Barrett–Joyner–Halenda (BJH) techniques (Fig. 8a). The BET surface area of the PPy-SnO_2_ NCs was 24.14 m^2^ g^−1^, indicating a moderate surface area that favours possible adsorption applications. The nitrogen adsorption–desorption isotherm exhibited a type IV shape with an H3 hysteresis loop typical of mesoporous materials.

BJH analysis of the desorption branch gave a total pore volume of 0.033 cm^3^ g^−1^ and an average pore diameter of 3.9 nm, which lies at the micro–mesopore boundary (Fig. 8b). The t-plot analysis indicated negligible true microporosity, suggesting that the accessible surface is dominated by narrow mesopores and external surface area, both of which are beneficial for the adsorption of dye molecules.

Overall, such porosity facilitates the diffusion and interaction of the target molecules with the nanocomposite, increasing its adsorption potential and catalytic use. These surface properties agree with the already reported mesoporous polymer-metal nanocomposites.^51^

## 4. Antioxidant studies

### 4.1. DPPH (2,2-diphenyl-1-picrylhydrazyl) assay

**Fig. 9:**
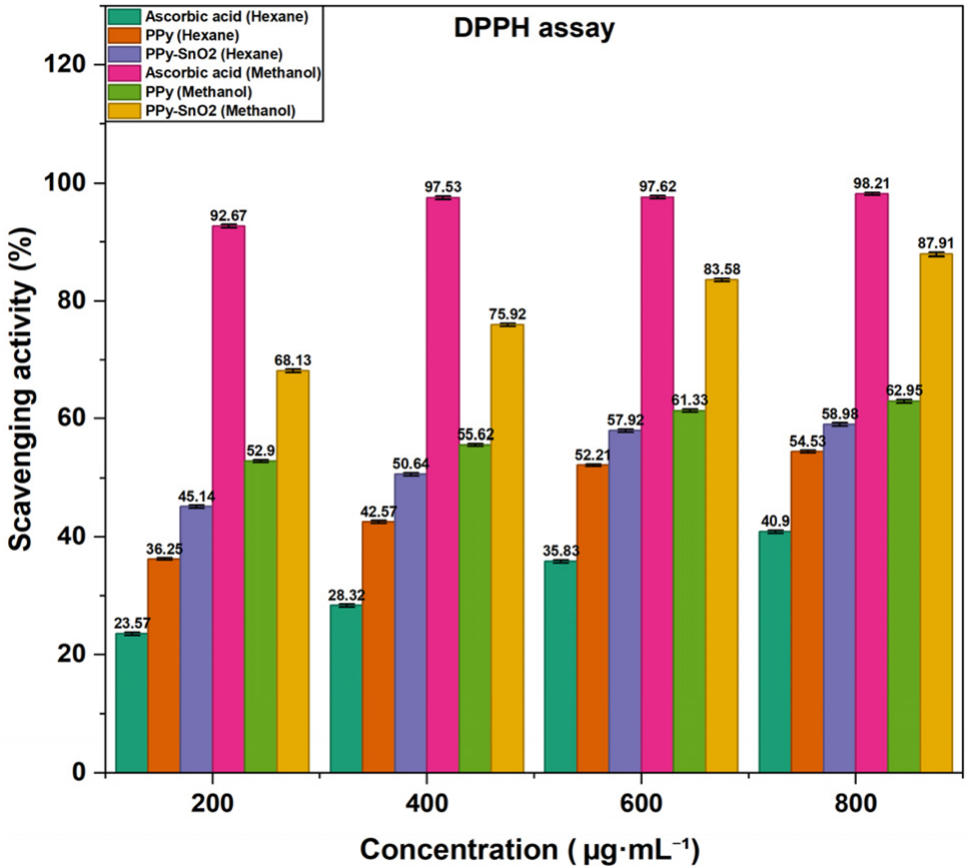
Graphical representation of scavenging activity by DPPH.

The DPPH radical scavenging method is an effective approach for determining antioxidant potential, interacting mainly through electron transfer with an additional addition *via* hydrogen atom transfer. In this study, antioxidants reduce the violet-coloured DPPH radical to a pale-yellow form, and a reduced absorbance at 517 nm indicates the level of radical scavenging. As shown in Fig. S9 (SI), the DPPH radical is neutralized by abstracting a hydrogen atom from the antioxidant (AH), resulting in the formation of the reduced DPPH-H and an antioxidant radical (A˙). All antioxidant measurements were assessed using PPy and PPy-SnO_2_ nanocomposites(NCs). Notably, *Foeniculum vulgare* extract was not studied for the assay medium, only washed and dried PPy and PPy-SnO_2_ NCs were examined to avoid any contribution from residual phytochemicals. In the current study, PPy and PPy-SnO_2_ nanocomposites demonstrated concentration-dependent antioxidant activity in methanol and hexane, with significantly greater effectiveness observed in methanol due to better nanomaterial dispersion and stronger interactions with radicals in polar media (Fig. 9). Ascorbic acid showed the highest activity, reaching 98.21 ± 1.14% in methanol, while PPy and PPy-SnO_2_ NCs achieved 62.95 ± 1.13% and 87.91 ± 1.06% inhibition, respectively, at 800 µg mL^−1^.

Each assay was performed three times (*n* = 3), and values are provided as mean ± SD. The consistently higher scavenging activity of PPy-SnO_2_ NCs when compared with PPy can be associated with the catalytic role of SnO_2_ nanoparticles, which promote electron transfer. These findings, consistent with previous studies,^52,53^ confirm that polymer–metal nanocomposites exhibit enhanced antioxidant capacity and hold promise as effective ROS scavengers.

The Scavenging activity was evaluated by the given equation:
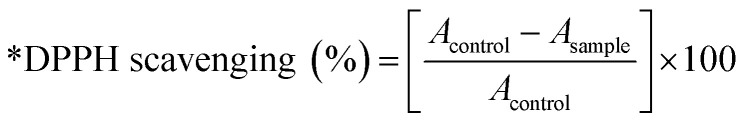


### 4.2. ABTS (2,2-azino-bis-3-ethylbenzothiazoline-6-sulphonic acid) assay

The antioxidant activity of PPy and PPy-SnO_2_ nanocomposites was further examined by the ABTS + cation radical approach. In this experiment, the blue-green ABTS + solution decolors upon interaction with antioxidants, as indicated by a decrease in absorbance at 734 nm (Fig. S11).

**Fig. 10:**
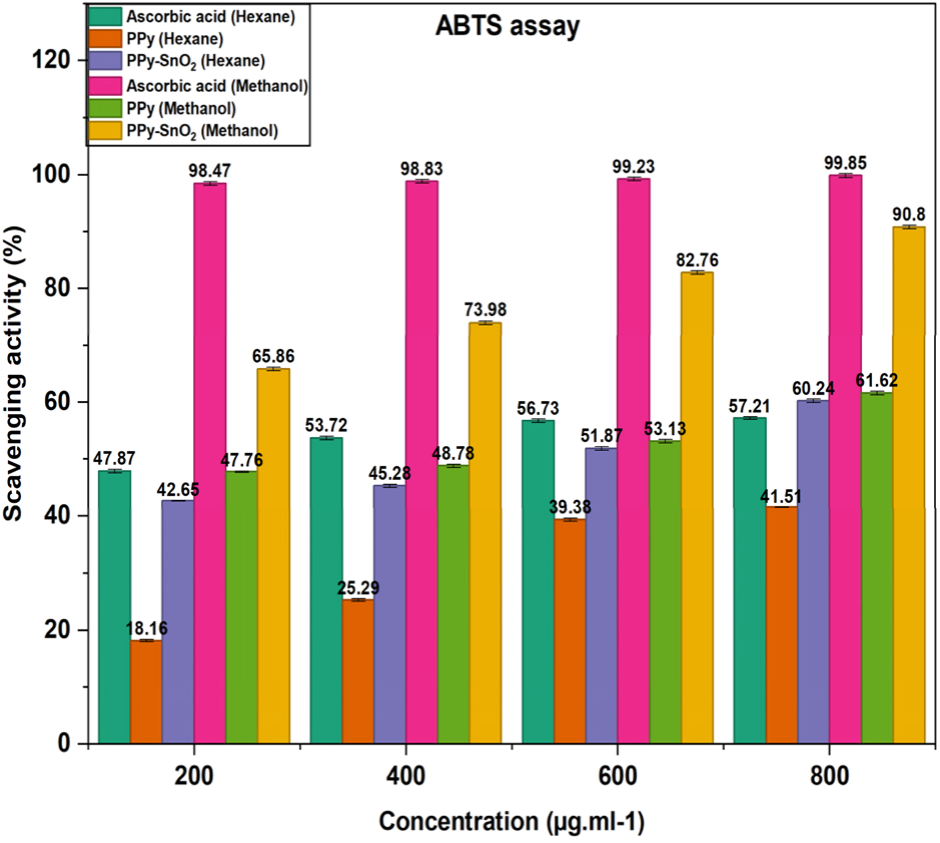
Graphical representation of scavenging activity by ABTS.

Both PPy and PPy-SnO_2_ NCs observed an increase in radical scavenging activity with varying the concentration range over the 200–800 µg mL^−1^ concentration range (Fig. 10), at 800 µg mL^−1^, PPy-SnO_2_ NCs reached 90.80% inhibition in methanol compared to 57.21% for PPy, whereas in hexane the values were 60.24% and 41.51%, respectively. Ascorbic acid, used as the standard, showed the most significant inhibition in methanol at 98.21 ± 1.14%. A similar trend was observed with the DPPH method, confirming its lower activity in hexane than in methanol and highlighting the key role of solvent polarity in nanocomposite–radical interactions.^52^ The percentage inhibition was determined by the equation:
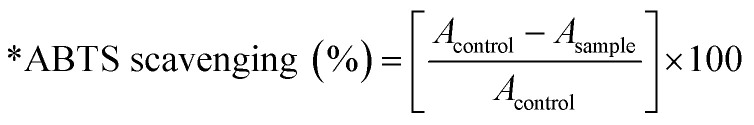


PPy-SnO_2_ NCs exhibited considerable antioxidant activity, which makes them emerging materials for various applications such as food packaging, biomedical coatings and water treatment, where the dual function of effective pollutant removal and radical scavenging is extremely favourable. However, their performance when compared with ascorbic acid, suggesting that they can support traditional antioxidants but cannot completely replace ascorbic acid.^36^

## 5. Polypyrrole-tin oxide nanocomposites for CV dye removal from aqueous solutions

The increasing contamination of the aquatic ecosystem by synthetic dyes from textile, pharmaceutical, and other industries has raised significant environmental concerns due to their toxicity, persistence, and resistance to biodegradation. Crystal violet is a commonly employed cationic triphenylmethane dye which is particularly resistant to conventional wastewater treatment. To address this concern, the adsorption performance of PPy-SnO_2_ nanocomposites was examined using CV, a model cationic dye, to evaluate their potential for environmental remediation. Batch studies were performed under static conditions to examine the impact of NC dose, dye concentration, time, and temperature on removal efficiency. All batch adsorption experiments were performed using washed and dried PPy-SnO_2_ nanocomposites as the adsorbent, without addition of *Foeniculum vulgare* to the adsorption medium. UV-visible spectrophotometry was employed to monitor the adsorption performance, quantified in terms of percentage removal (%*R*) and equilibrium adsorption capacity (*Q*_e_), evaluated by following equations:
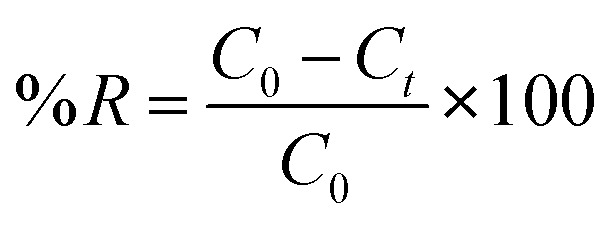

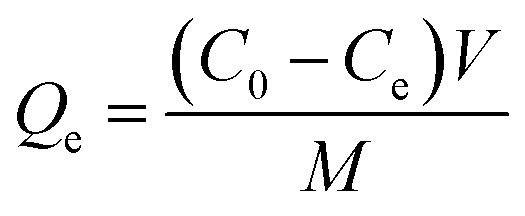
*C*_0_ represents the initial concentration (mg L^−1^), *C*_*t*_ represents the final concentration of dye at time *t* (mg L^−1^), *C*_e_ is the equilibrium concentration of dye (mg L^−1^), *Q*_e_ is the equilibrium adsorption capacity (mg g^−1^), and *V* denotes to the volume of solution used through the experiment (*L*), *M* refers to mass of adsorbent (*g*).

### 5.1. Effect of parameters on removal of dye

#### 5.1.1. Effect of adsorbent dose

**Fig. 11:**
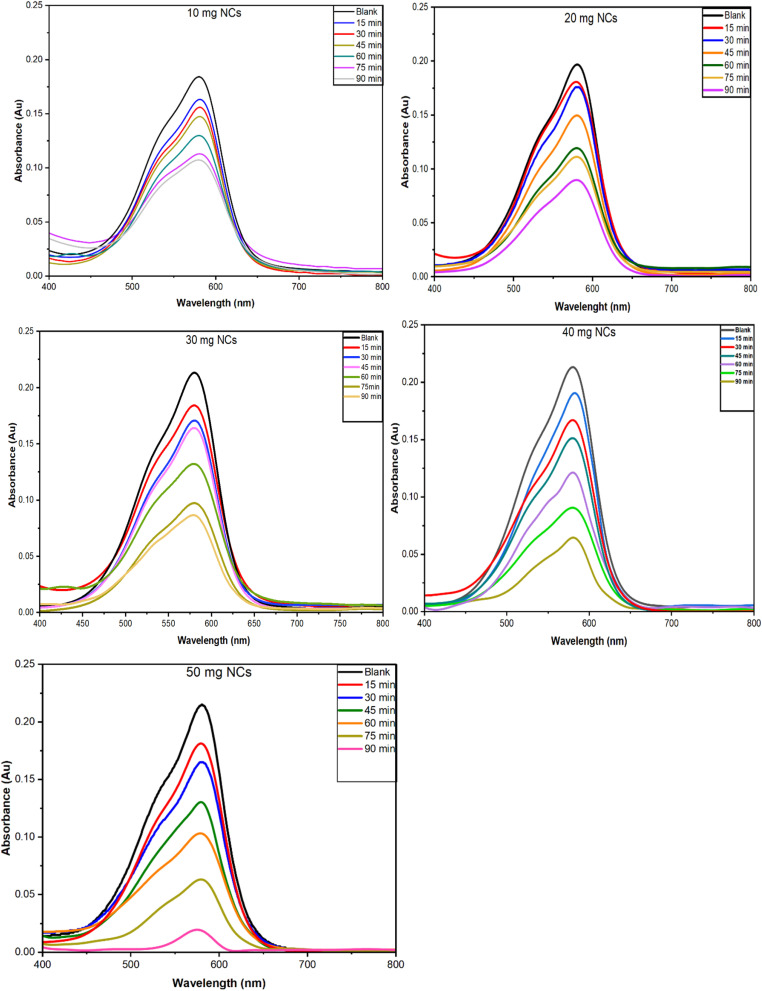
The effect of the dose of PPy-SnO_2_ NCs (10–50 mg) on the removal of dye at pH 7, initial dye concentration of 10 ppm, and contact time of 90 minutes.

The dosage of PPy-SnO_2_ NCs significantly influenced the dye removal efficacy. In this study, adsorption was performed by changing the dose of NCs from 10 to 50 mg under maintained conditions (pH 7 and stirring speed of 1200 rpm). As illustrated in Fig. 11, a dose of 10 mg resulted in lower dye removal, due to less adsorption sites. The increase in NC dose from 20 mg to 40 mg indicates moderate dye removal efficiency, whereas dye removal efficacy reached its optimum at an adsorbent dosage of 50 mg. This is resulting from more vacant sites and larger surface area that enhances adsorption capacity. No saturation effect was observed, but higher doses can cause aggregation, which highlights the need of optimizing the adsorbent dose.^54^ This demonstrates that dye removal efficiency is strongly dose-dependent, with higher PPy-SnO_2_ NCs providing greater adsorption performance.

#### 5.1.2. Effect of pH

**Fig. 12:**
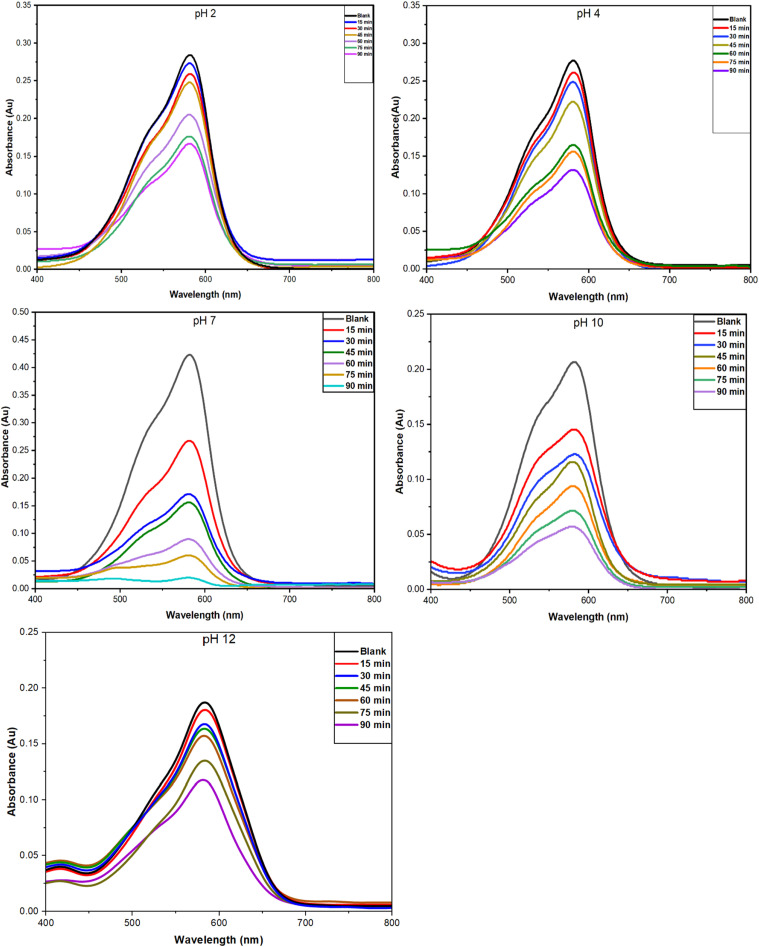
The effect of pH on dye removal efficiency of PPy-SnO_2_ NCs with 10 ppm dye solution, with 50 mg of NCs and contact time of 90 minutes.

The batch study of CV on PPy-SnO_2_ NCs was accessed across a pH range of 2 to 12 with a fixed NC dose of 50 mg, a dye concentration of 10 ppm at room temperature. The highest dye removal was observed at neutral pH (7), whereas acidic (pH 2–4) and alkaline (pH 10–12) conditions significantly reduced dye removal efficiency (Fig. 12). The point of zero charge (pH_p_zc) of PPy-SnO_2_ NCs was found as 6.8, which corresponds to the pH where the net surface charge of the NCs is negligible. Below this pH_p_zc value, protonation of the PPy-SnO_2_ NCs surface induced electrostatic repulsion with the cationic CV dye and thereby reduced adsorption. Above this pH_p_zc value, the surface of PPy-SnO_2_ NCs is negatively charged which favours CV dye adsorption. However, adsorption decreases at high pH due to OH^−^ ion competition and mobility of dye aggregates is decreased under basic conditions. At neutral pH (7), near the pH_p_zc, electrostatic interactions are minimal, allowing van der Waals and π–π interactions, resulting in maximum adsorption efficiency. The outcomes suggest the significant role of pH in maintaining the interactions among dye and PPy-SnO_2_ NCs, with neutral conditions offering effective adsorption.^55^

#### 5.1.3. Effect of concentration

**Fig. 13:**
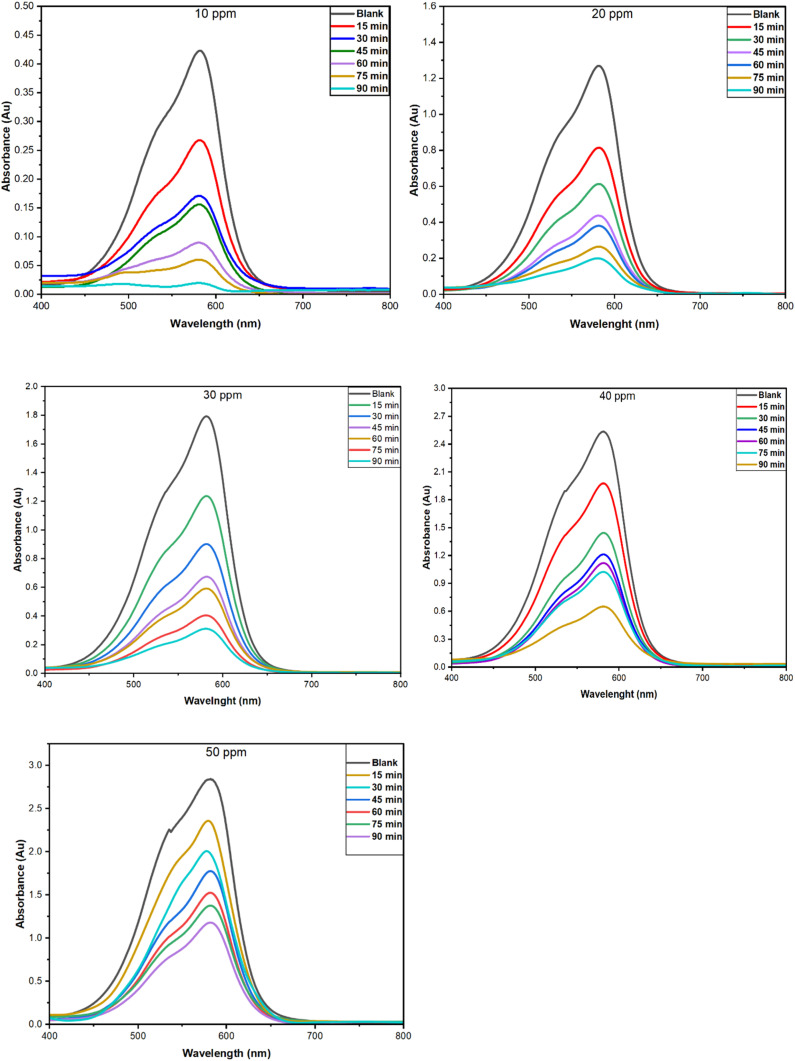
The effect of initial dye concentration on removal efficiency by PPy-SnO_2_ NCs at a pH solution of 7 and 50 mg of NCs in a contact time of 90 min.

An adsorption study was performed using initial CV dye concentrations ranging from 10 to 50 ppm, with 50 mg of NCs and pH 7 at room temperature for 90 minutes. Fig. 13 demonstrates that the removal efficacy increased with a decrease in dye concentration, reaching its optimum at 10 ppm. At higher concentrations, the adsorption efficiency was reduced, suggesting that the surface of the nanocomposite has reached a saturation level and binding sites were limited. This data indicates that the dye adsorption is strongly impacted by the balance between number of dye molecules and the available active sites, highlighting the need to select a suitable initial concentration for effective dye removal.

#### 5.1.4. Effect of temperature

**Fig. 14:**
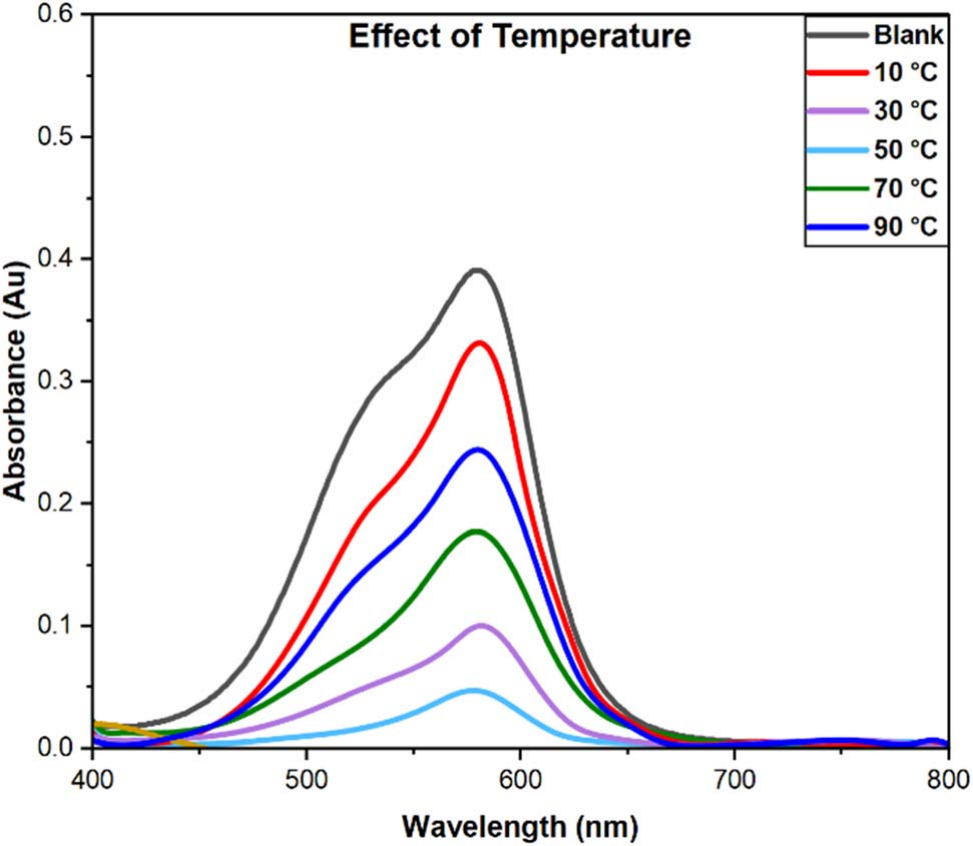
Temperature-dependent adsorption of dye was performed with fixed condition: a dye concentration of 10 ppm, 50 mg of NCs, a pH of 7 and a 90-min contact time.

The temperature-dependent behavior of dye adsorption by PPy-SnO_2_ NCs was examined across a range of 10–90 °C, with 50 mg of NCs at pH 7, 10 ppm dye in 90 minutes. As illustrated in Fig. 14, the removal efficiency increased with a rise in temperature, indicating its optimum at 50 °C, and then decreased at 70 °C and 90 °C. The initial rise in adsorption efficiency at moderate temperatures is due to faster dye-molecule movement and improved accessibility of active sites. However, above 50 °C, removal efficiency decreases, likely due to desorption and weaker interactions between dye and PPy-SnO_2_ NCs, showing that high temperatures reduce adsorption stability.^56^ The thermodynamic studies were evaluated to gain insight into the adsorption. The equilibrium constant (*K*_c_) was evaluated in dimensionless form using the equation *K*_c_ = *C*_ads_/*C*_e_

Where *C*_ads_ denotes the adsorbed dye molar concentration on the nanocomposites at equilibrium, the free energy change (Δ*G*°) was evaluated from the equation Δ*G*° = −*RT* ln *K*_c_

*R* is the gas constant (8.314 J mol^−1^ K^−1^), *T* refers to absolute temperature (*K*), and *K*_c_ is the equilibrium constant. The positive Δ*H* = 38.53 kJ mol^−1^ indicates that adsorption is endothermic, while the positive Δ*S* = 121.87 J mol^−1^ K^−1^ suggests a rise in entropy at the substrate–liquid interface. Gibbs free energy (Δ*G*) shifts from 4.06 kJ mol^−1^ at 283 K to −6.00 kJ mol^−1^ at 363 K, indicating that adsorption becomes spontaneous at higher temperatures. The high correlation coefficient (*R*^2^ value = 0.995) from the Van't Hoff plot supports the reliability of these thermodynamic data.^57^

#### 5.1.5. Effect of time

**Fig. 15:**
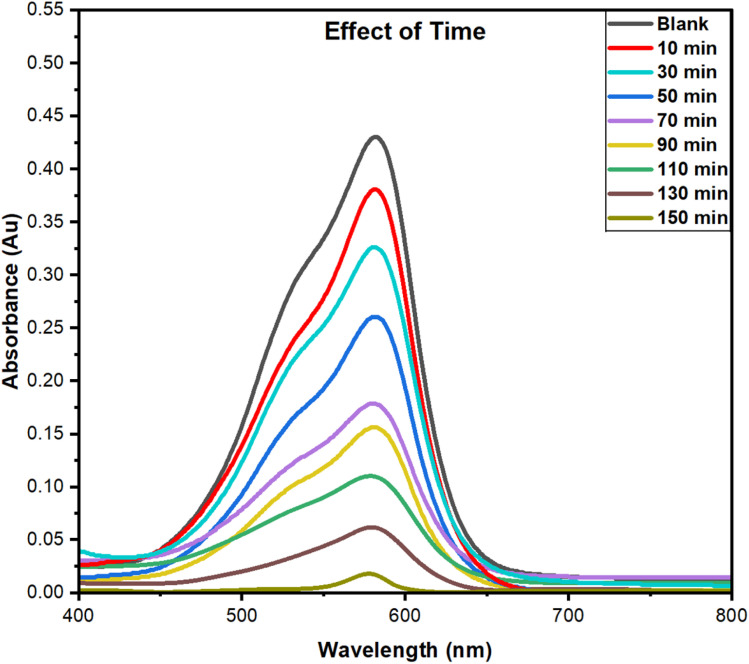
The effect of contact time was examined with fixed conditions: a dye concentration of 10 ppm, NCs dose of 50 mg, a pH of 7, and a temperature of 50 °C.

The study on the dye adsorption on the PPy-SnO_2_ nanocomposite was investigated over a contact time range of 10–150 minutes, as illustrated in Fig. 15. The adsorption experiment was performed using a 10 ppm dye concentration, at neutral pH, and NCs dose of 50 mg, with a constant stirring rate of 1200 rpm at 50 °C.^36^ As depicted in Fig. 15, removal efficacy enhances with longer contact times, reaching a plateau after approximately 150 minutes. These results show that this process is time-dependent, as longer contact times enable more dye molecules to bind with the available active sites. The initial rapid adsorption is resulting from the abundance of unoccupied sites, while the slower approach to equilibrium after 150 minutes suggests site saturation.^58^

## 6. Kinetic and adsorption isotherms

The adsorption study of crystal violet onto PPy-SnO_2_ NCs was accessed through kinetic and isotherm studies. This analysis provided a detailed insight into adsorption mechanism, the dye-nanocomposite surface interaction, and the equilibrium characteristics, thereby confirming the efficiency and reliability of the nanocomposites for dye removal.

### 6.1 Adsorption isotherm

The adsorption equilibrium of crystal violet by PPy-SnO_2_ NCs was analyzed to understand the adsorption mechanism and surface characteristics. The adsorption isotherm demonstrates how dye molecules are balanced among the aqueous solution and the adsorbent at equilibrium. The adsorption study was accessed employing the Langmuir and Freundlich isotherms.

#### 6.1.1. Langmuir isotherm

The equilibrium adsorptive removal of CV dye was initially analysed using the Langmuir isotherm, which assumes monolayer adsorption on a homogeneous substate with finite, equivalent sites and no interactions between adsorbed molecules. The linearized Langmuir equation is expressed as:
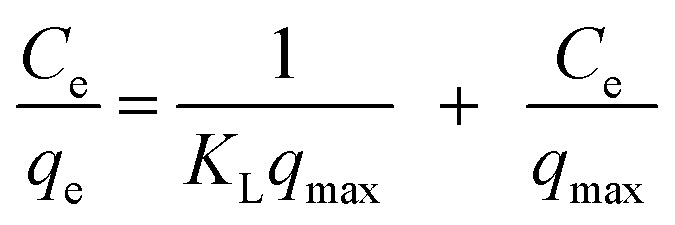
where *q*_max_ represents the maximum monolayer adsorption capacity (mg g^−1^), and *K*_L_ is the Langmuir constant, which indicates the affinity between the adsorbent and dye molecules.

**Fig. 16:**
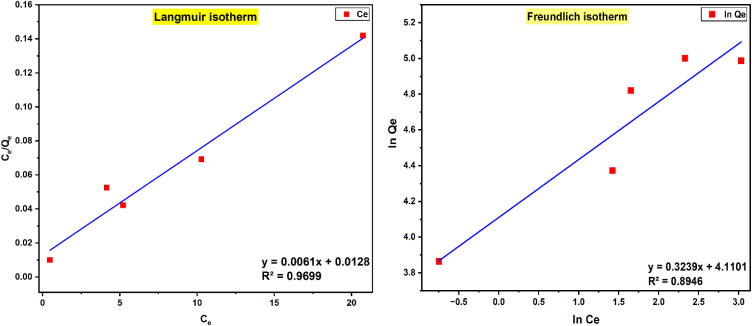
Langmuir and Freundlich isotherm model fitting curve for the CV dye adsorptive removal onto synthesized PPy-SnO_2_ NCs.

The results were correlated with the linearised Langmuir equation (*C*_e_*versus Q*_*e*_), resulting in a high correlation coefficient (*R*^2^ ≈ 0.9698), indicating excellent agreement with the model and confirming monolayer adsorption as the dominant mechanism (Fig. 16a). This excellent agreement ensures that CV dye removal take place on the defined solid adsorption sites of the PPy-SnO_2_ nanocomposites rather than through homogeneous solution-phase reactions. The maximum adsorption capacity (*Q*_max_) was determined from the slope of the Langmuir plot, yielding 162.6 mg g^−1^, highlighting the excellent performance of PPy-SnO_2_ NCs in removing crystal violet dye molecules from aqueous solutions. Furthermore, the Langmuir constant (*K*_L_) was found to be 0.48 L mg^−1^, suggesting a higher binding affinity of the CV molecules for the NCs surface.^59^ The favourability of the adsorption process was further evaluated by calculating the dimensionless separation factor (*R*_L_). The calculated *R*_L_ values for all tested concentrations are within the 0–1 range, indicating that the removal of the CV dye by PPy-SnO_2_ NCs surface is favourable and involves significant adsorbent–adsorbate interactions.

The results demonstrate that CV dye molecules effectively interact with the uniform binding sites on the PPy-SnO_2_ NCs surface, likely by π–π interactions, electrostatic attractions, and hydrogen bonding. The excellent adsorption capacity of nanocomposites and the rapid attainment of equilibrium highlight their potential for efficient cationic dye removal in aqueous media.

#### 6.1.2. Freundlich isotherm

To investigate reaction pathways and surface heterogeneity, the equilibrium results were examined by the Freundlich approach, which highlights multilayer adsorption on a non-uniform surface and allows interactions between adsorbed molecules. The Freundlich equation in linear form is represented byln *q*_e_ = ln *K*_F_ + (1/*n*) ln *C*_e_

*K*_F_ represents the Freundlich isotherm parameters, and 1/*n* refers to heterogeneity factor, which indicates the adsorption intensity. The linear fitting of ln *Q*_e_ against ln *C*_e_ yielded a slope value of 0.3239 (1/*n* = 0.3239, *n* = 3.09) and an intercept of 4.1101, corresponding to a *K*_F_ = 60.9 mg g^−1^ (L g^−1^ (L mg^−1^)^1/*n*^, as shown in Fig. 16b. The value of Freundlich constants (*K*_F_ and *n*) confirmed the favourability of adsorption, with an *n* value ranging from 1 to 10, signifying strong dye-NCs surface affinity. However, this model yielded slightly lower *R*^2^ values (0.8946) than the Langmuir model, suggesting heterogeneity in the PPy-SnO_2_ system. The high *K*_F_ value further indicates the significant adsorption capacity of the synthesized nanocomposites.^60^ This model represents adsorption occurring on heterogeneous sites and is not limited to formation of monolayer.

### 6.2. Adsorption kinetics

This study was investigated to understand the efficiency of Crystal Violet (CV) dye removal and the mechanisms governing adsorption on PPy-SnO_2_ nanocomposites. Adsorption behaviour was evaluated by Kinetic models and determined whether the mechanism involved physisorption or chemisorption. The pseudo-1st-order and 2nd-order model are employed to study adsorption kinetics and to understand adsorption mechanisms.

#### 6.2.1. Pseudo-1st-order model

**Fig. 17:**
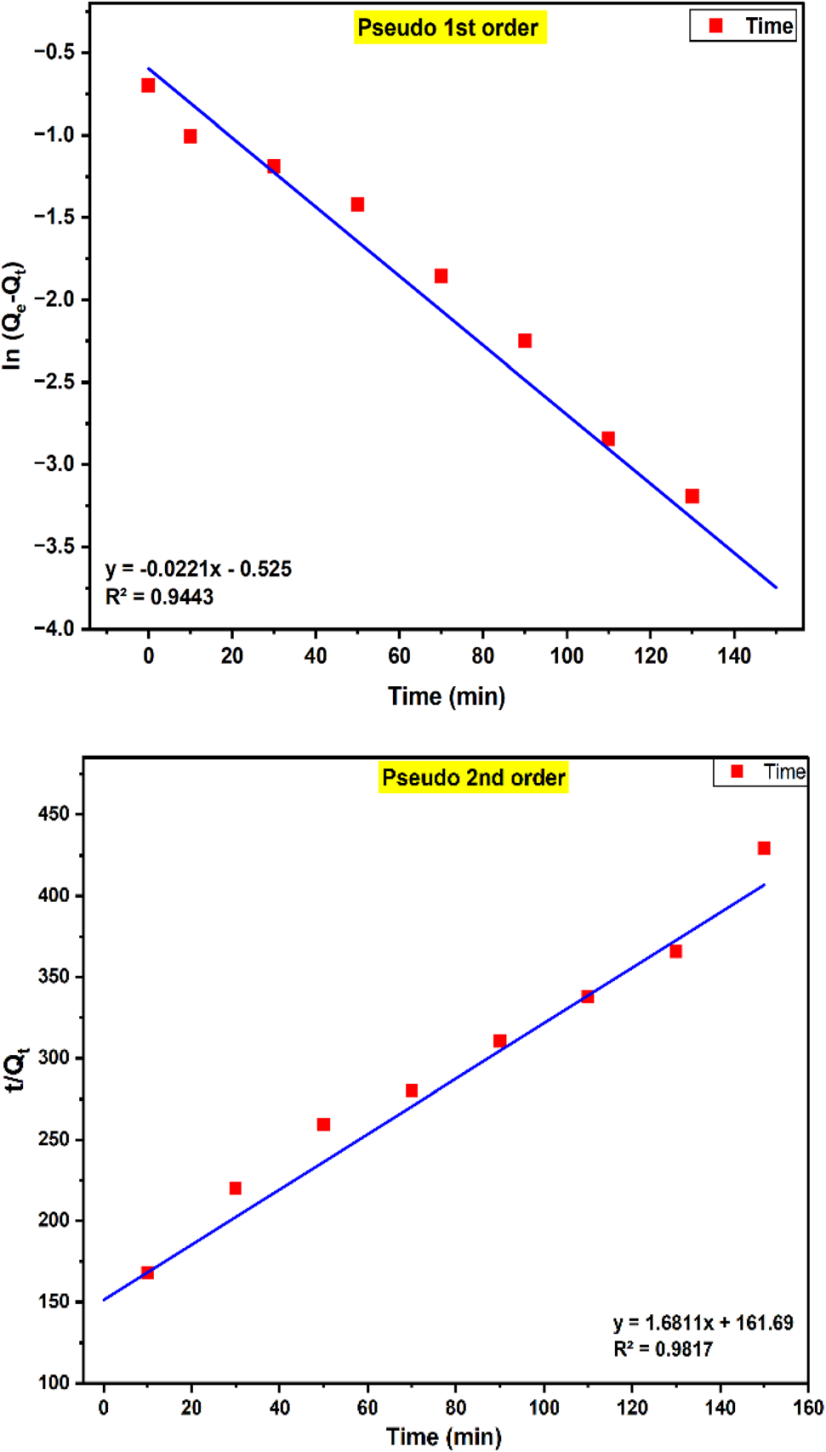
The plots illustrating the pseudo 1st-order and pseudo-2nd-order kinetics for crystal violet adsorption onto PPy-SnO_2_ NCs.

This approach is based on the assumption that the adsorption rate depends on the availability of binding sites on the adsorbent surface, suggesting physical adsorption (physisorption; Fig. 17a). The linearized form of this model is given byln (*q*_e_ − *q*_*t*_) = ln *q*_e_ − *k*_1_*t*Here, *k*_1_ (min^−1^) represents the pseudo-1st-order rate constant. These parameters were estimated from intercept and slope, respectively, of the linear plot of ln (*q*_e_ − *q*_*t*_) against *t*. Although the pseudo-1st-order model yielded linear plots for the adsorption of dye molecules using synthesized NCs, the *q*_e_ value determined from the kinetic model did not match the experimental data. Additionally, the correlation coefficients (*R*^2^ = 0.9443) were lower than those obtained for pseudo-2nd-order kinetics. This data indicates that the pseudo-1st-order model, which primarily describes physisorption, does not sufficiently capture the adsorption process. The findings demonstrates that physisorption is not the major mechanism. However, the process is most likely due to chemisorption, which involves stronger interactions among the dye molecules and the functional sites on the PPy-SnO_2_ surface.^61,62^

#### 6.2.2. Pseudo-second-order model

This model states that the adsorption rate is governed by the square of the vacant active sites and depends on the amount of adsorbate adsorbed at a given time (*q*_*t*_) and at equilibrium (*q*_e_). This indicates that the adsorption mechanism is dominated by chemisorption. The model is expressed as:
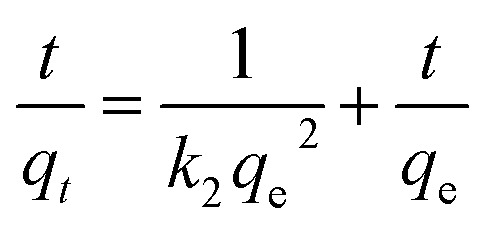


The pseudo-2nd-order provided a better fit to the experimental adsorption data for removal of dye by PPy-SnO_2_ nanocomposites. The equilibrium adsorption capacities (*q*_e_) predicted by this model closely matched the observed experimental data having a correlation coefficient (*R*^2^) of 0.998, indicating an excellent fit (Fig. 17b). These observations suggest that electron–exchange interactions among the dye molecules and reactive functional sites on the nanocomposite's surface primarily control adsorption.^63^ The detailed isotherm and kinetics parameters that include constants, calculated adsorption capacities and correlation coefficients are given in Table S5 (SI file).

## 7. Mechanism of CV dye adsorption onto PPy-SnO_2_ nanocomposites

**Fig. 18:**
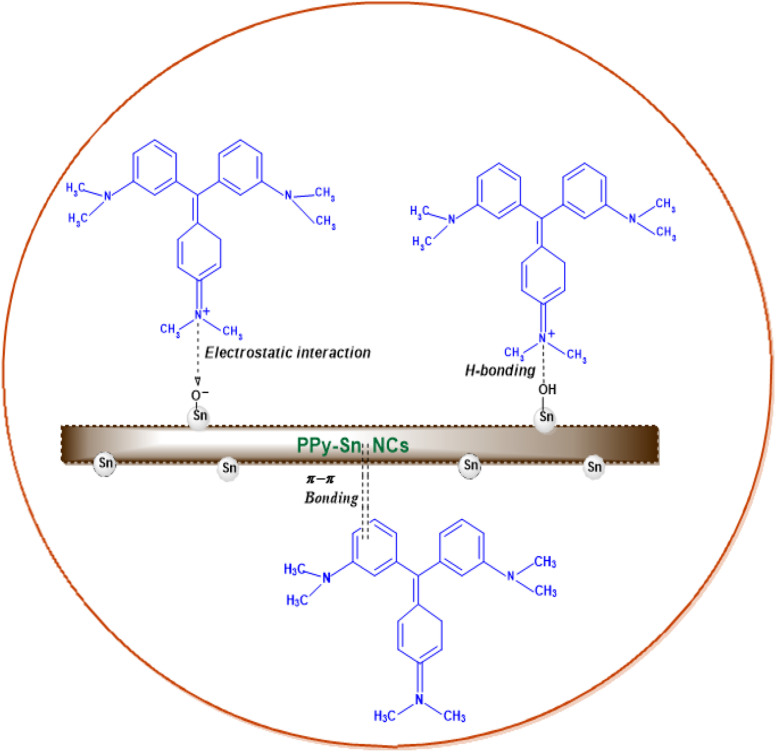
Adsorption of crystal violet on PPy-SnO_2_ nanocomposites *via* electrostatic, hydrogen-bonding, and π–π interaction.

The adsorptive removal of CV dye onto PPy-SnO_2_ NCs occurs through a synergistic combination of hydrogen bonding, electrostatic interactions and π–π stacking among the conjugated rings of PPy and the CV dye molecules. At neutral pH, the cationic Crystal Violet (CV) dye molecules are electrostatically attracted to negatively charged oxygen sites of SnO_2_. At the same time, –OH groups on the surface of nanocomposites participate in hydrogen bonding with dye molecules. The incorporated SnO_2_ NPs offer more active sites within the PPy matrix, which enhances dye adsorption and interfacial interaction. This cooperative mechanism results in efficient dye removal from aqueous solutions. A representation of the proposed adsorption mechanism is shown in Fig. 18, highlighting the key interactions responsible for the high adsorption performance of nanocomposite.

## 8. Comparative analysis of crystal violet adsorption performance

To evaluate the efficiency of the synthesized PPy-SnO_2_ nanocomposites, a comparison was made with previously reported adsorbents used for crystal violet (CV) removal. The PPy-SnO_2_ NCs exhibited 92% removal efficiency under optimized conditions (pH 7, 10 ppm dye concentration, 50 mg adsorbent dose and 50 °C within 150 min), showing adsorption capacity (*q*_max_) of 162.6 mg g^−1^ followed by the Langmuir isotherm model. Kinetic analysis confirmed that this study followed pseudo-2nd-order equation, suggesting chemisorption as the dominant mechanism. The adsorption capacities and experimental conditions of the already reported adsorbents are summarised in Table 3. The findings suggests that the *q*_max_ of synthesized PPy-SnO_2_ NCs is comparable to various nanomaterial-based adsorbents.

**Table 3:** Comparison table for CV dye adsorption performance of PPy-SnO_2_ NCs with reported adsorbents

Adsorbent	*Q*_max_ (mg g^−1^)	Optimum pH	Temperature (°C)	Contact time (min)	Reference
PPy-SnO_2_	162.6	7	50	150	[our study]
Ferrite-biochar composite	325.4	7–8	30	120	64
Modified carbon spheres	134.6	6	25	180	65
Carbon-sphere/Titania-nanotube composite (TNTs@Cs)	84.7	5.5	25–30	180	66
Natural clay	203.6	7	25	120	67

## 9. Conclusions

This study successfully synthesized PPy-SnO_2_ nanocomposites by incorporating *Foeniculum vulgare* extract-derived SnO_2_ nanoparticles into polypyrrole synthesized *via* oxidative polymerization. Comprehensive characterization by UV-vis, FTIR, DLS, zeta potential, SEM-EDS, HRTEM and XRD confirmed the successful formation, stability and uniform incorporation of SnO_2_ within the PPy matrix. The nanocomposites showed excellent adsorption performance toward crystal violet, achieving 92% removal under optimized conditions (pH 7, 10 ppm dye, 50 mg PPy-SnO_2_, 50 °C, 150 min) with a maximum adsorption capacity of 162.6 mg g^−1^, underscoring their suitability for dye removal applications. In addition, they exhibited significant antioxidant activity in methanol at 800 µg mL^−1^, with 87.90% and 90.80% radical scavenging in the DPPH and ABTS assays, respectively, and higher efficiencies than in hexane. These results demonstrate that PPy-SnO_2_ nanocomposites are promising dual-functional materials that effectively combine high-efficiency dye removal with potent radical scavenging, thereby holding considerable potential for environmental remediation. To the best of our knowledge, this is among the first reports on *Foeniculum vulgare*-assisted PPy-SnO_2_ nanocomposites that integrate efficient crystal violet adsorption with solvent-dependent antioxidant activity. This sustainable green synthesis approach can be further tailored for use in medical, agricultural and environmental sectors. Future work may focus on optimizing the nanocomposite formulation, exploring reusability and stability under realistic conditions, and extending their application to other relevant pollutants and reactive oxygen species, thereby advancing the role of green nanotechnology in sustainable development.

## Author contributions

Conceptualization, methodology, software, writing – original draft preparation, P. K.; resources, project administration, data curation, R. S.; supervision, validation, formal analysis, investigation, writing – review and editing, R. B.; A. P.; all authors have read and agreed to the published version of the manuscript.

## Conflicts of interest

The authors declare no conflicts of interest.

## Supplementary Material

RA-016-D5RA09693F-s001

## Data Availability

The data supporting this article have been included in the supplementary information (SI) and as part of the manuscript. Supplementary information: additional experimental and plant extract details, along with characterization data (Fig. S1–S19 and Tables S1–S6) supporting the results presented in this manuscript. See DOI: https://doi.org/10.1039/d5ra09693f.
