# Supplementary material for: Green synthesis of polypyrrole-SnO2 nanocomposites using Foeniculum vulgare extract for crystal violet adsorption and solvent-dependent radical scavenging
Source: RSC Adv. 2026 Feb 3;16(8):6967–84. doi: 10.1039/d5ra09693f (PMC12865685; doi:10.1039/d5ra09693f)
Supplement: RA-016-D5RA09693F-s001 [file RA-016-D5RA09693F-s001.pdf]

Supplementary Information (SI) for RSC Advances.
This journal is © The Royal Society of Chemistry 2026

Supplementary Information

Green *Foeniculum vulgare* Assisted Synthesis of Polypyrrole–SnO_2 Nanocomposites: Dual Function in Crystal Violet Adsorption and Solvent-Dependent Radical Scavenging

Priya Kaushik[a], Ruchi Bharti[a*], Renu Sharma[a], and Annu Pandey[b*]

[a]Department of Chemistry, University Institute of Sciences, Chandigarh University, Punjab, India

[b]Department of Fibre and Polymer Technology, KTH Royal Institute of Technology, Stockholm, Sweden

*Corresponding authors E-mail addresses: ruchi.uis@cumail.in (R. Bharti), annua@kth.se (A. Pandey).

Plant Material and Extract Preparation:

Foeniculum vulgare seeds(fennel) were purchased from a local herbal market in Chandigarh, India. As *F. vulgare* is a commonly available culinary and medicinal plant, the seeds were used without further authentication. The species is well established; therefore, no special collection permits or ethical approval were required.

Result:

Characterization: The polypyrrole-coated stannous nanocomposites (PPy–SnO_2 NCs) employed in this study were synthesized via chemical oxidative polymerization. The synthesized PPy-SnO_2 NCs were characterized by various analytical techniques. Fourier transform infrared (FTIR) spectra were conducted on a PerkinElmer FTIR spectrometer, and UV-Vis absorption spectra were monitored using a Shimadzu UV-1900 spectrophotometer. The morphological features and elemental analysis were accessed via scanning electron microscopy with energy-dispersive spectroscopy (SEM-EDS, SU8010 series, HITACHI). Particle size and zeta potential were analysed with a Zetasizer Nano (Malvern Analytical, UK) provided with dynamic light scattering, and TEM-EDS was performed using a CRYO ARM™ 300 II instrument.

UV-VIS:

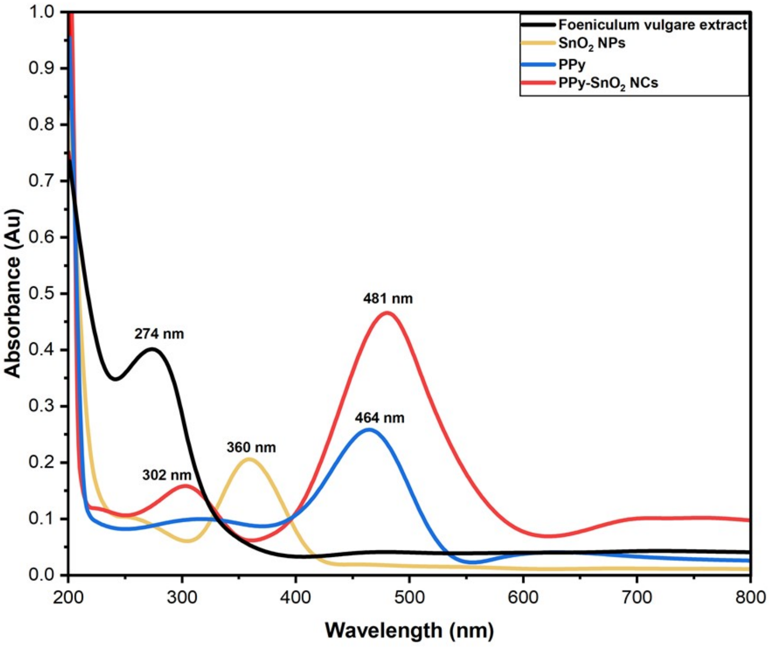

Figure 1: UV spectrum of (a) Foeniculum vulgare extract (b) Polypyrrole (c) SnO_2 nanoparticles (d) PPy-SnO_2 NCs

FTIR Spectroscopy:

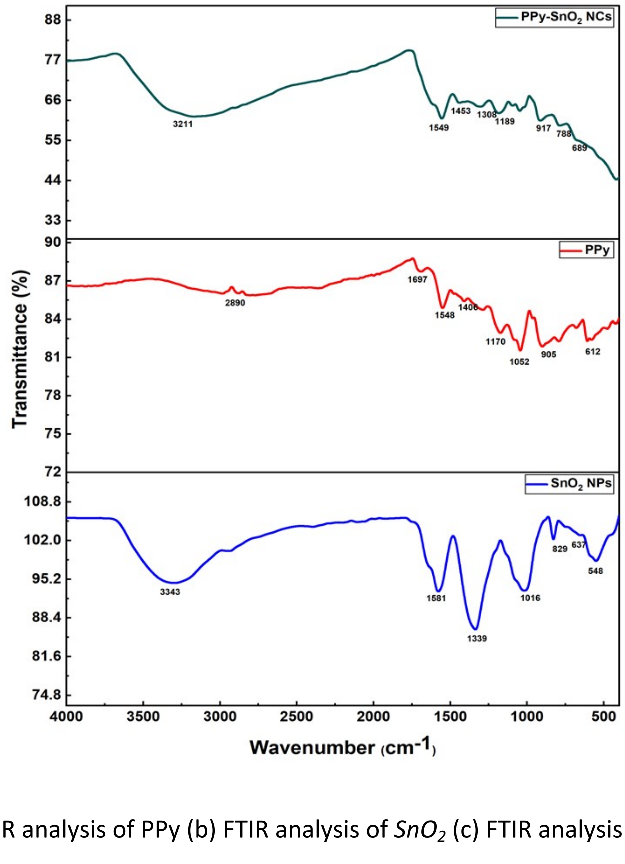

Figure S2: (a) FTIR analysis of PPy (b) FTIR analysis of *SnO_2* (c) FTIR analysis of PPy- SnO_2 NCs.

XRD:

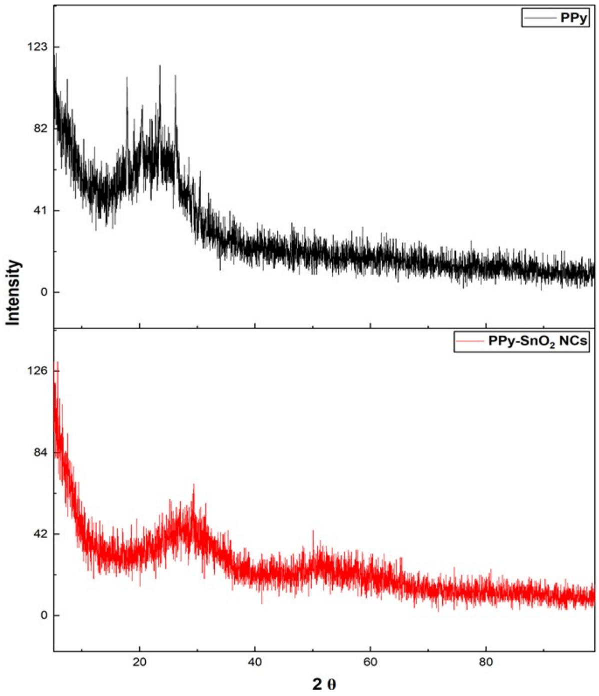

Figure S3: XRD analysis of Synthesized Ppy, and PPy-SnO_2 NCs.

SEM-EDS:

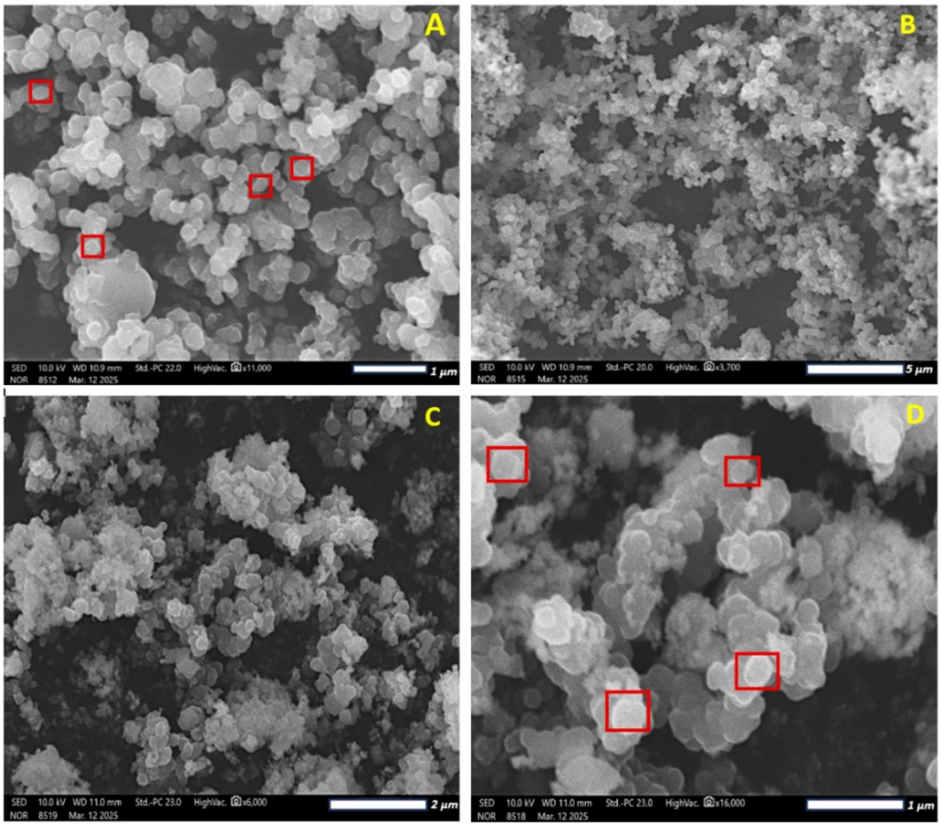

Figure S4a-d: SEM image of synthesized (a), (b) PPy and (c), (d) PPy-SnO_2 NCs at different magnification

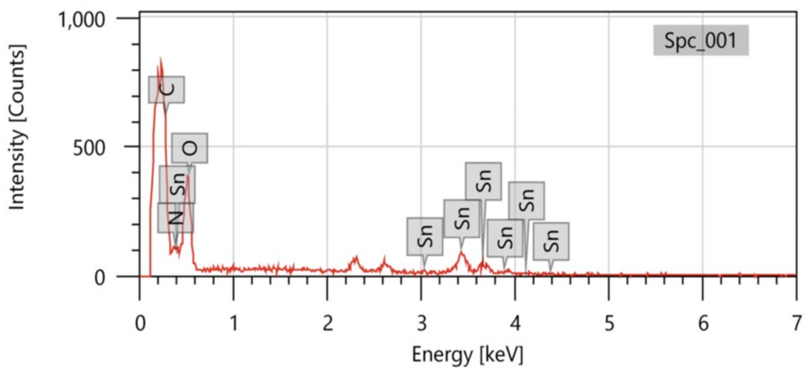

Figure S4(e): SEM-EDS of synthesized PPy-SnO_2 nanocomposites

HRTEM:

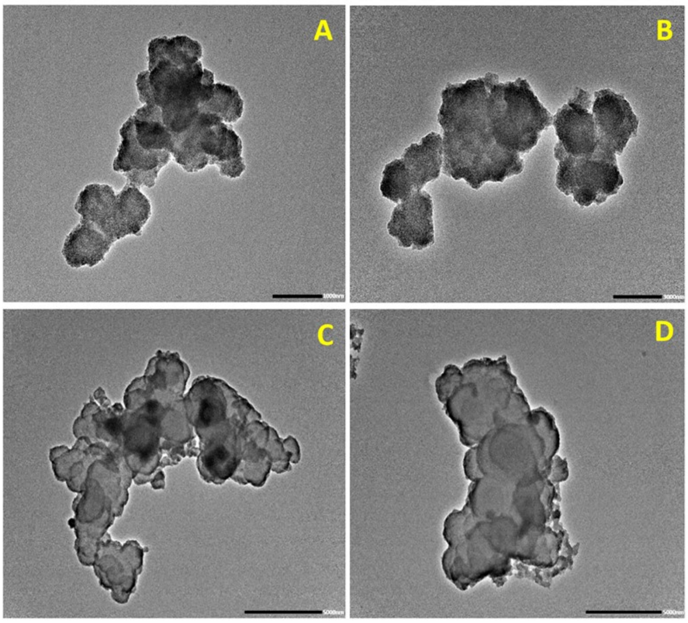

Figure S5: HRTEM image of synthesized (a), (b) PPy and (c), (d) PPy-SnO_2 NCs

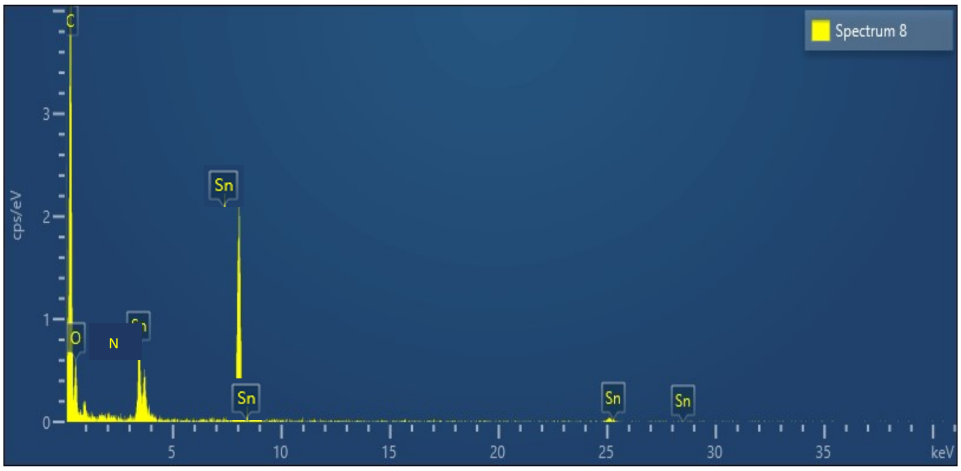

Figure S5 (e): HRTEM- EDS analysis of Synthesized PPy- SnO_2 Nanocomposites

DLS Analysis:

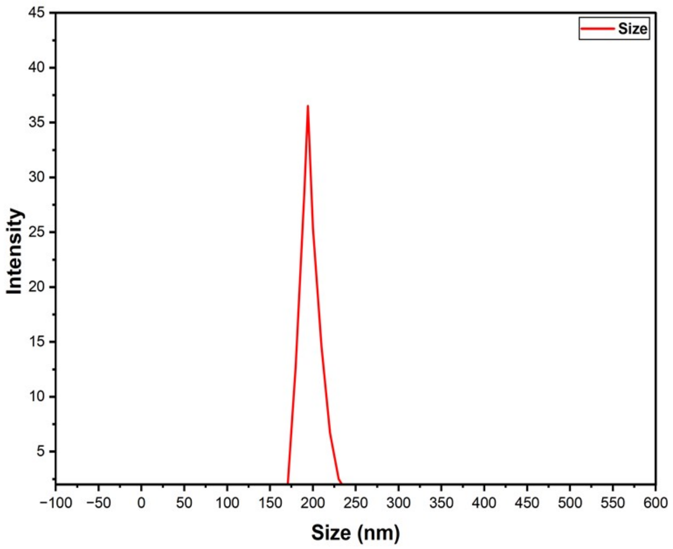

Figure S6: DLS analysis of Synthesized PPy- SnO_2 Nanocomposites

Zeta potential:

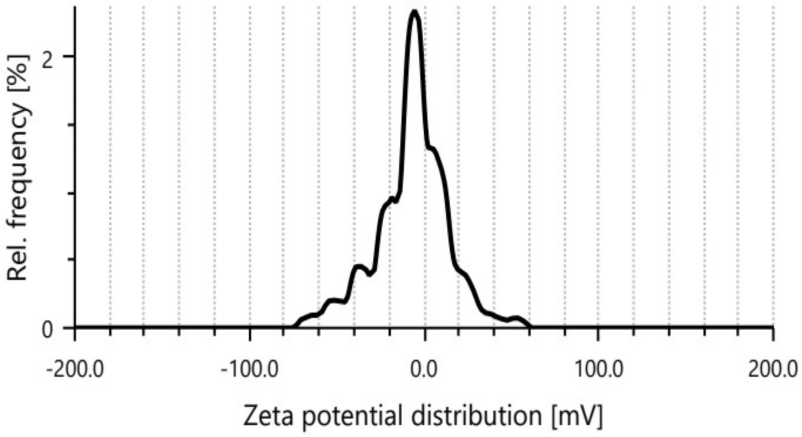

Figure S7: Zeta potential of PPy– SnO_2 nanocomposites, indicating surface charge and colloidal stability

BET analysis

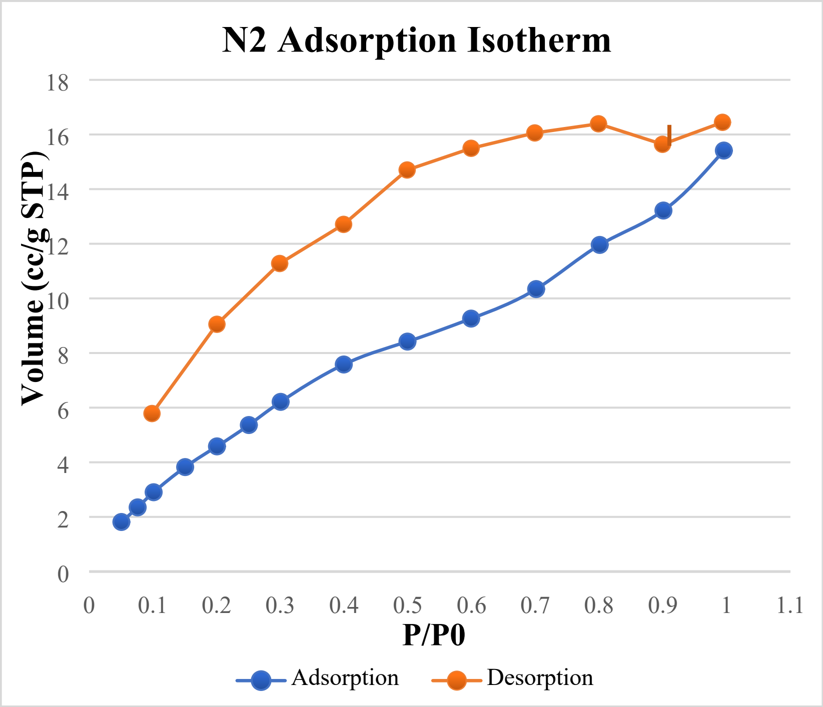

(a)

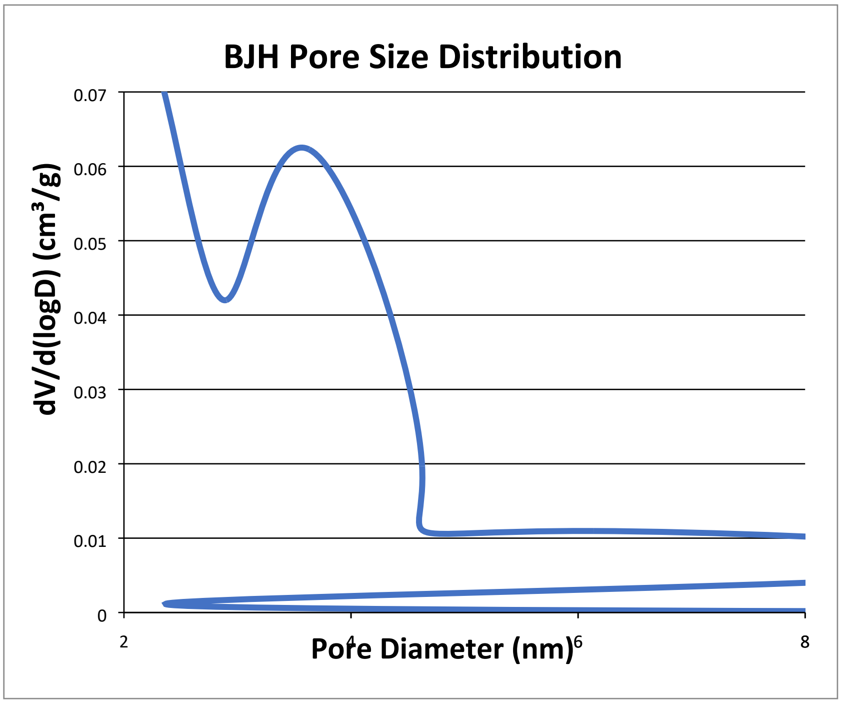

(b)

Figure S8: Nitrogen adsorption–desorption isotherm of PPy-SnO_2 NCs exhibiting Type IV behavior, characteristic of mesoporous structure, with a BET surface area of 24.14 m^2/g.

DPPH assay:

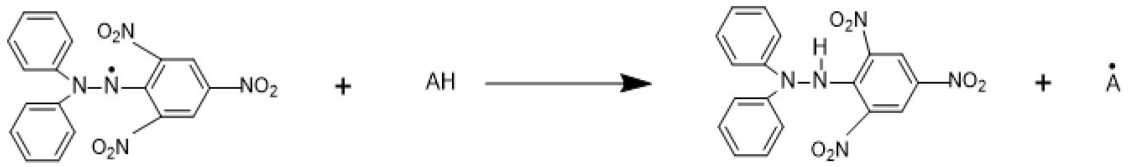

Figure S9: Mechanism for the radical scavenging activity of nanocomposites by antioxidant activity

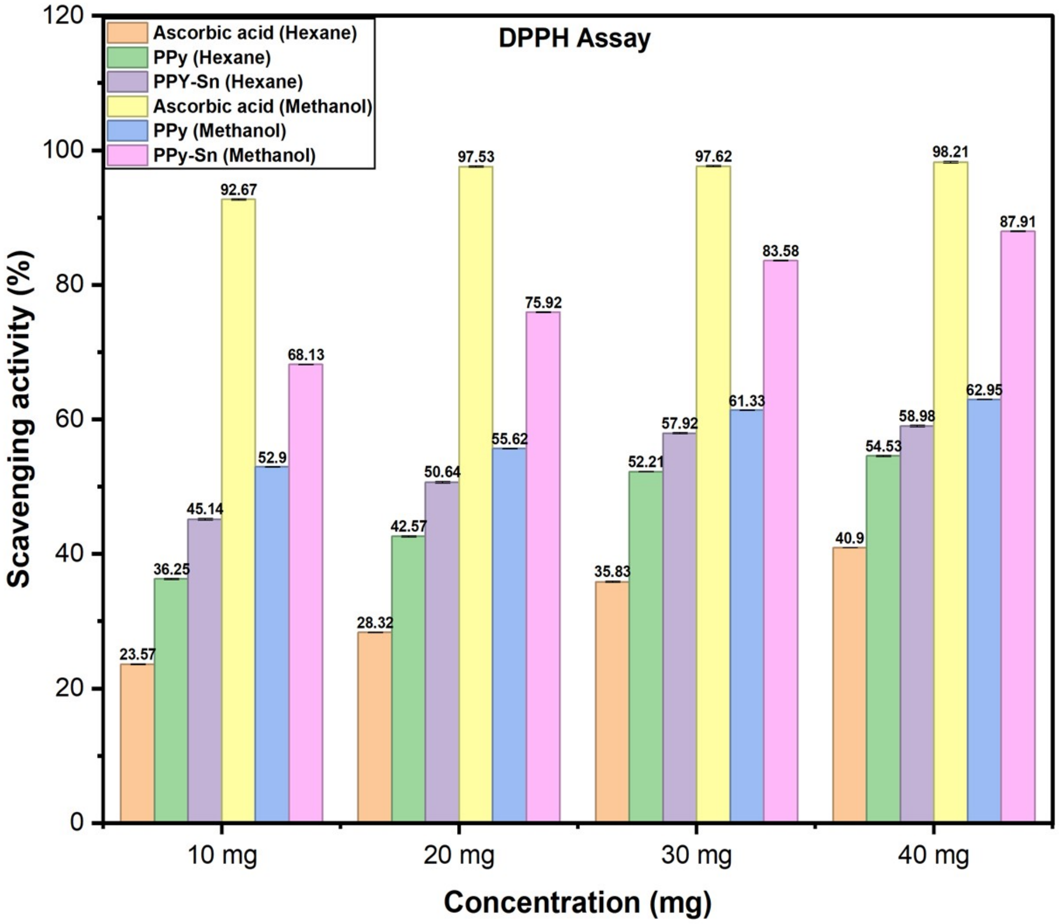

Figure S10: Graphical representation of Scavenging activity by DPPH

ABTS Assay:

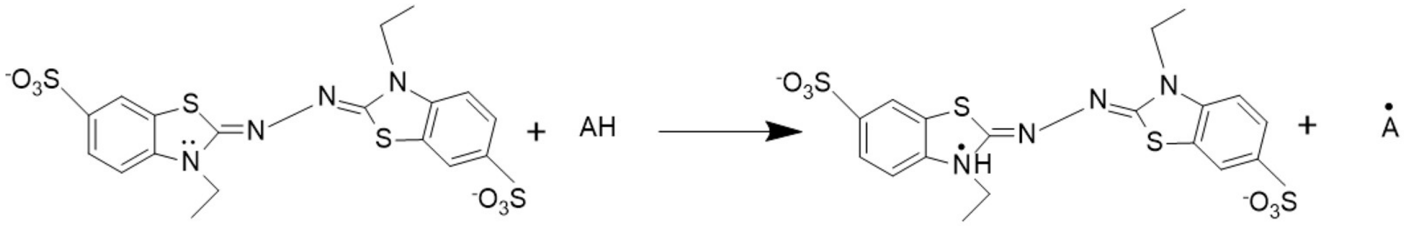

Figure S11: Chemical mechanism of the radical scavenging activity of nanocomposites by antioxidant activity (ABTS assay)

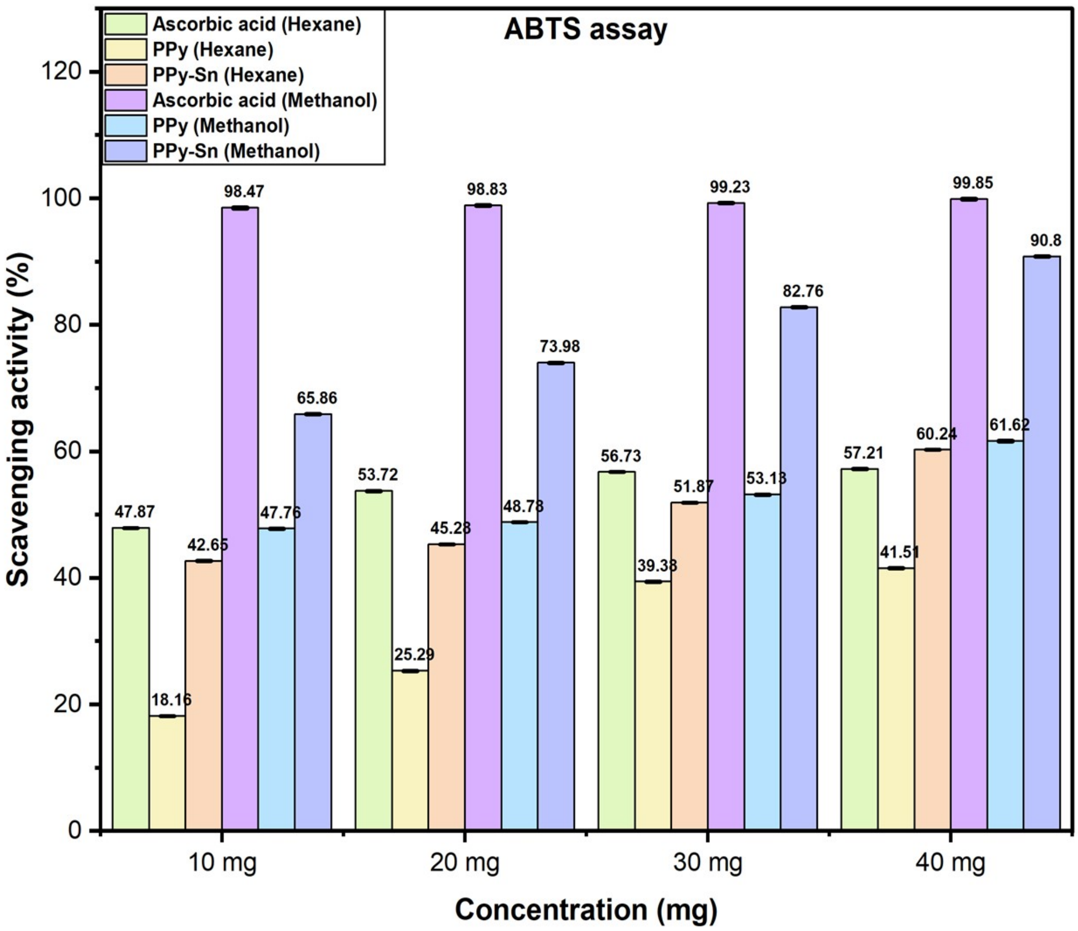

Figure S12: Graphical representation of Scavenging activity by ABTS

4.1.1. Effect of dose of NCs

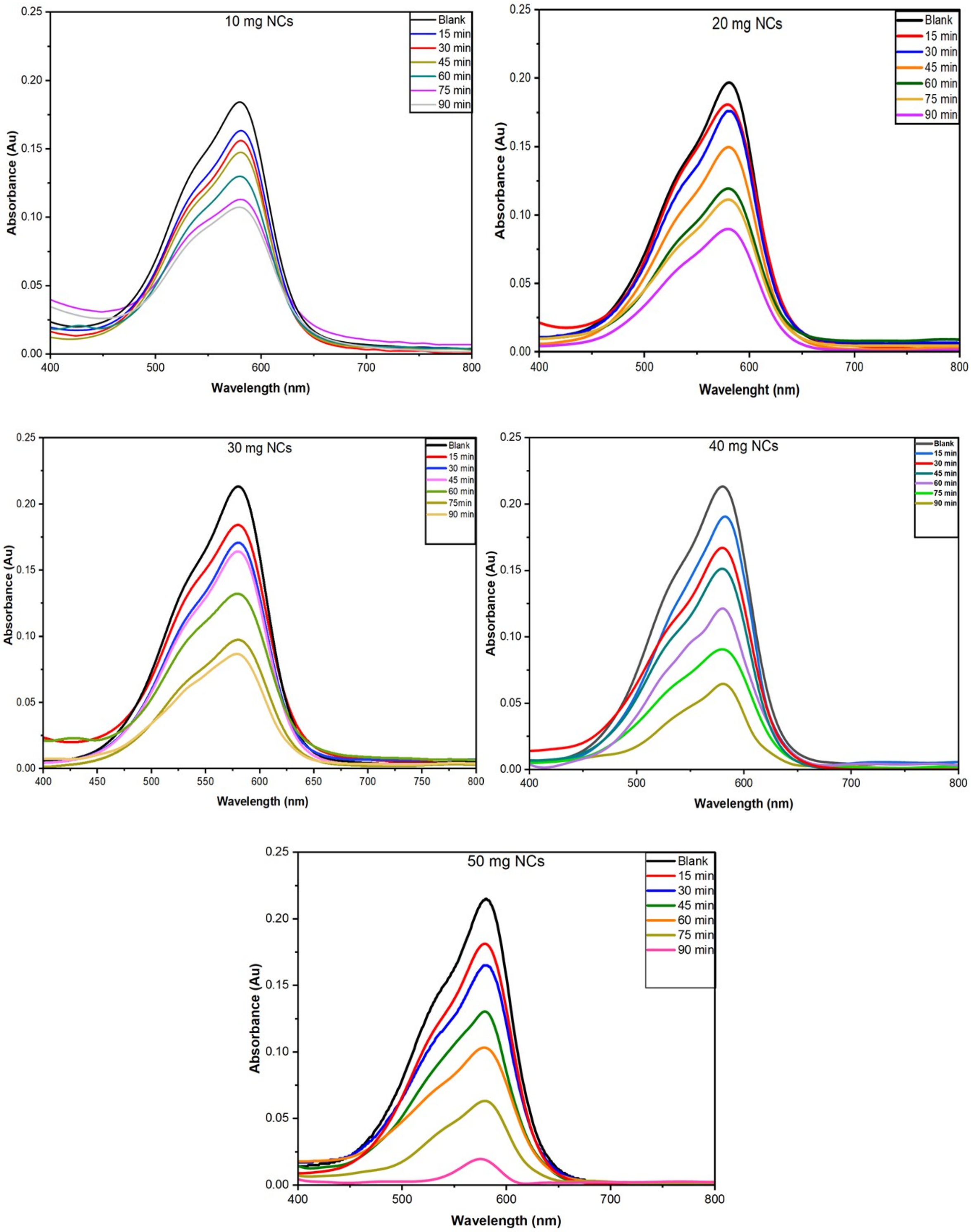

Figure S13: The Effect of dose of adsorbent (PPy- SnO_2 NCs) as 10 mg -50 mg on removal of Crystal violet dye.

4.1.2. Effect of pH:

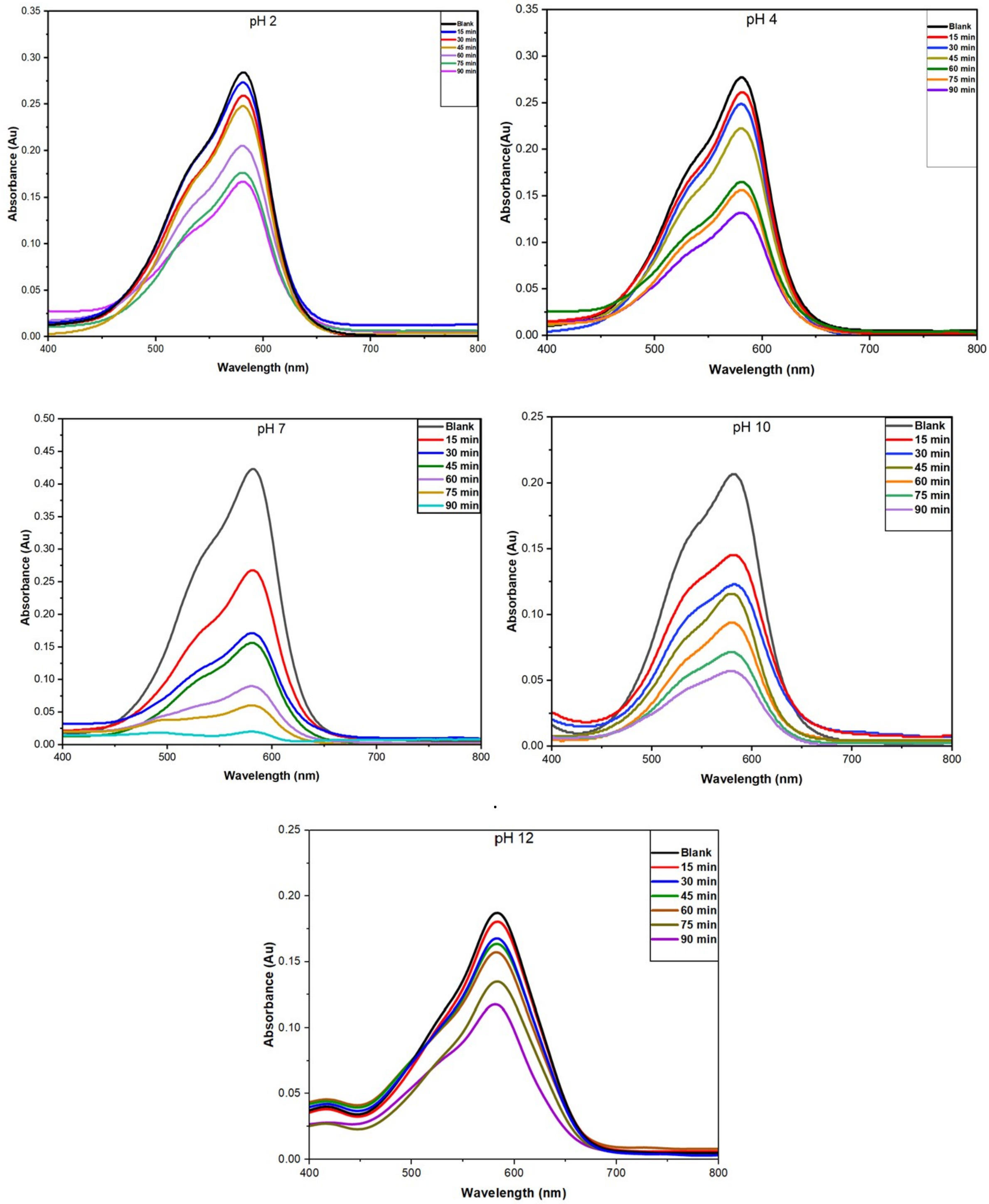

Figure S14: The effect of pH on dye removal was investigated under conditions of a dye solution concentration of 10 ppm, a contact time of 90 minutes, and an adsorbent dose of 50 mg.

4.1.3. Effect of Concentration:

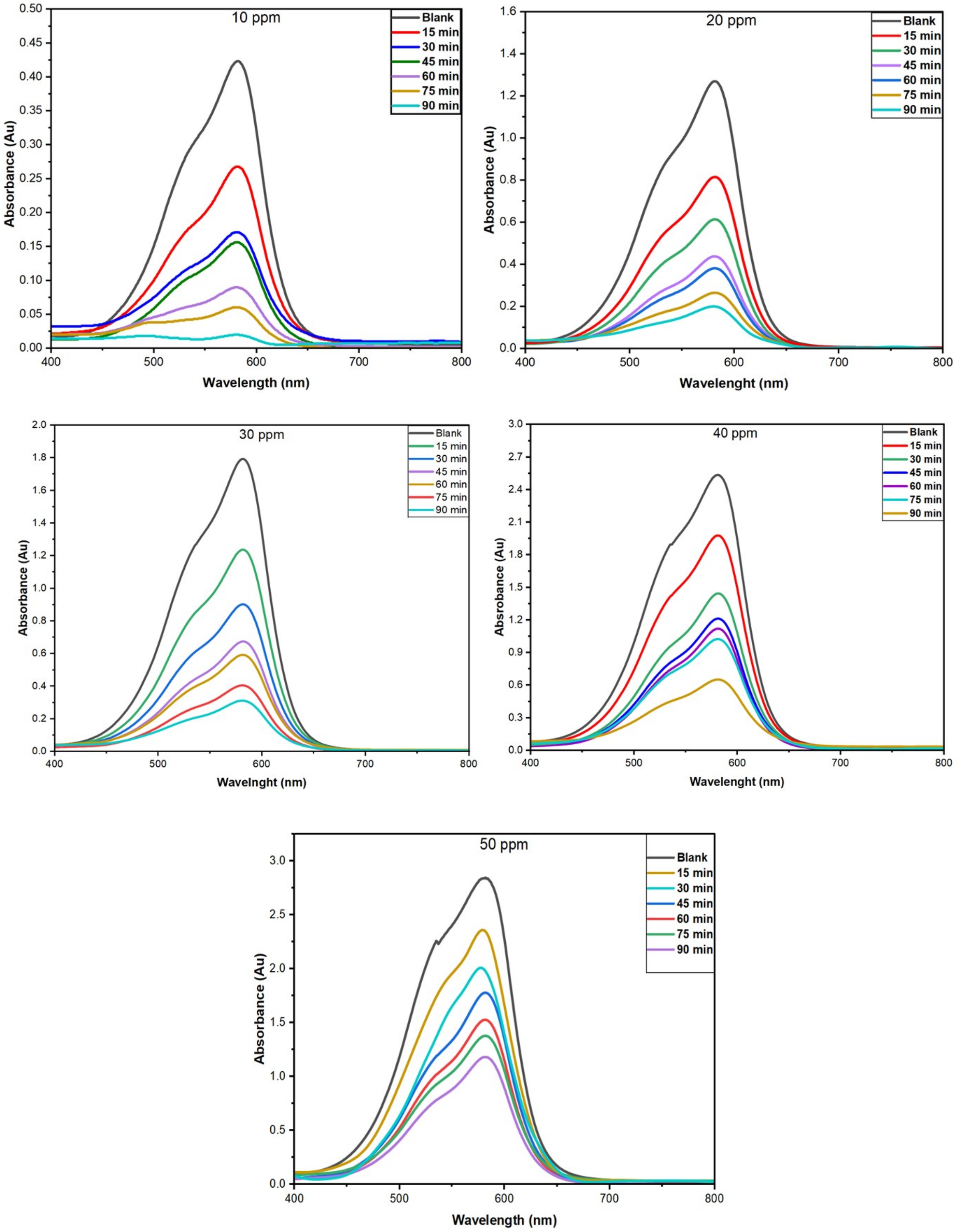

Figure S15: The effect of concentration dye on removal of dye was investigated under conditions of a dye solution concentration of 10 ppm, a contact time of 90 minutes, and an adsorbent dose of 50 mg.

4.1.4. Effect of Temperature:

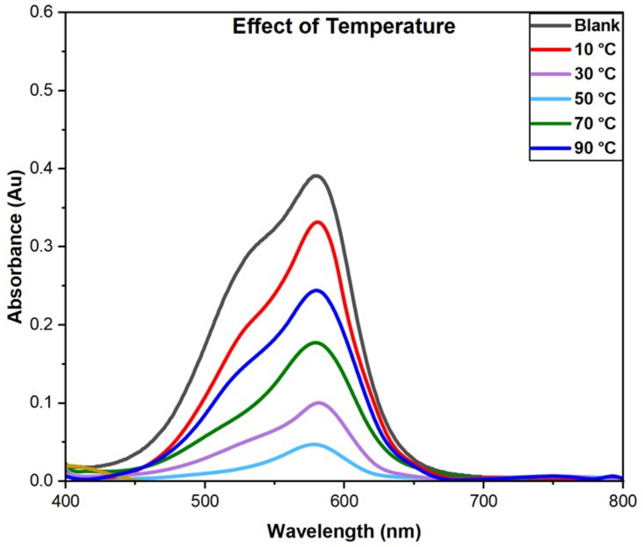

Figure S16: The effect of temperature on the removal of the dye was investigated under constant conditions, including a dye concentration of 10 ppm, an adsorbent dose of 50 mg, a pH of 7 in 90 minutes of contact time

4.1.5. Effect of Time:

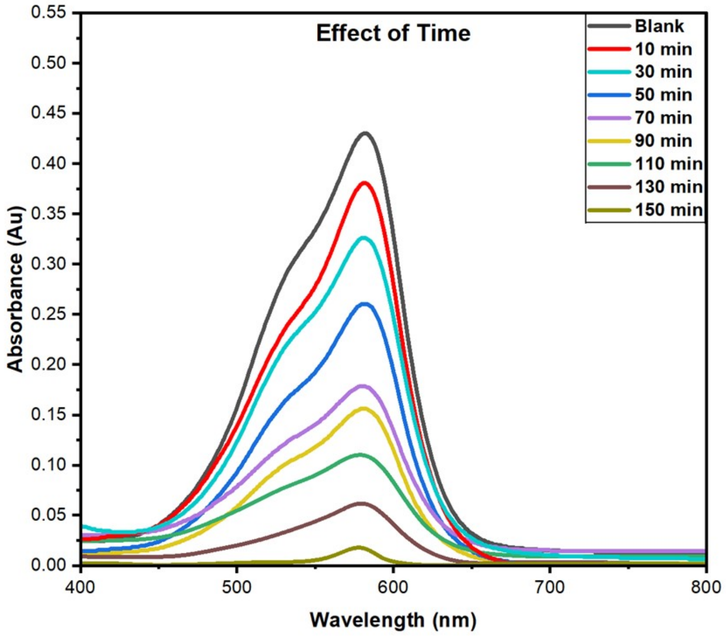

Figure S17: The effect of contact time on the removal of the dye was investigated under constant conditions, including a dye concentration of 10 ppm, an adsorbent dose of 50 mg, a pH of 7, and a temperature of 50°C.

6.1.1. Langmuir Isotherm:

The linearized Langmuir equation is expressed as:

$$\frac{C_e}{q_e} = \frac{1}{K_L q_{max}} + \frac{C_e}{q_{max}}$$

The maximum adsorption capacity (Q_{max}) was evaluated from the slope of the Langmuir plot using the following equation:

$$q_{max} = \frac{1}{Slope}$$

giving a value of 162.6 mg g^{-1}, highlighting the excellent performance of PPy-SnO_2 NCs in removing Crystal violet dye molecules from aqueous solutions. Furthermore, the Langmuir constant was obtained from the intercept according to

$$K_L = \frac{1}{Intercept * q_{max}}$$

Resulting in K_L =0.48 L/mg, suggesting strong binding affinity of CV molecules towards the nanocomposite surface.[59]

The favorability of the adsorption process was further assessed by calculating the dimensionless separation factor (R_L), which is defined by the Langmuir isotherm as:

$$R_L = \frac{1}{1 + K_L C_0}$$

Where C0 is the initial concentration, The calculated RL values across for all tested concentrations within the 0–1 range, confirming that the adsorption of CV dye onto PPy-SnO2 nanocomposites surface is favorable and involves significant adsorbent-adsorbate interactions.

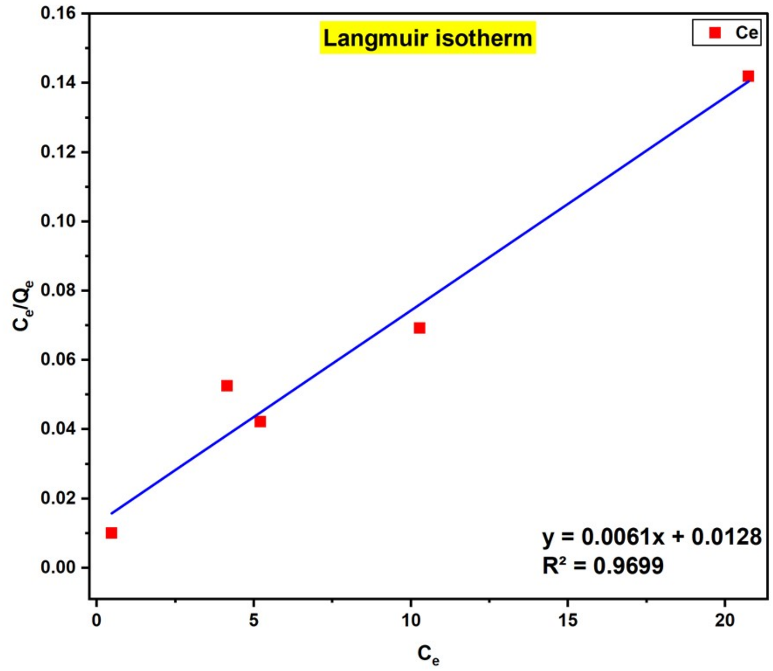

Figure S18a: Langmuir isotherm plot showing the adsorption of Crystal Violet dye onto synthesized PPy–SnO_2 nanocomposites.

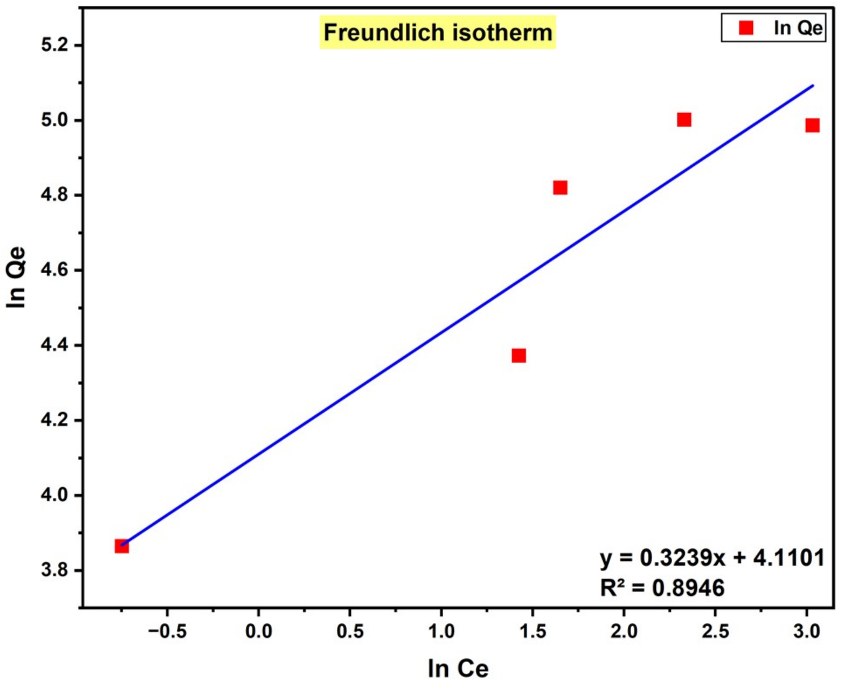

Figure S18b: Freundlich isotherm Plot for the removal of crystal violet dye by synthesized PPY-SnO_2 NCs.

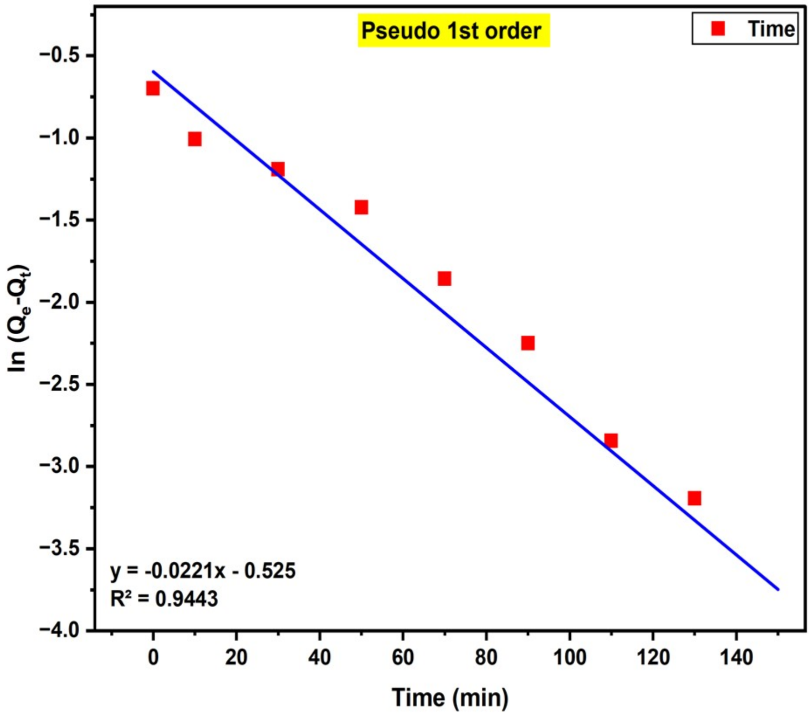

Figure S19a: The plot showing kinetics of Pseudo first-order for removal of crystal violet dye by PPy-SnO_2 NCs

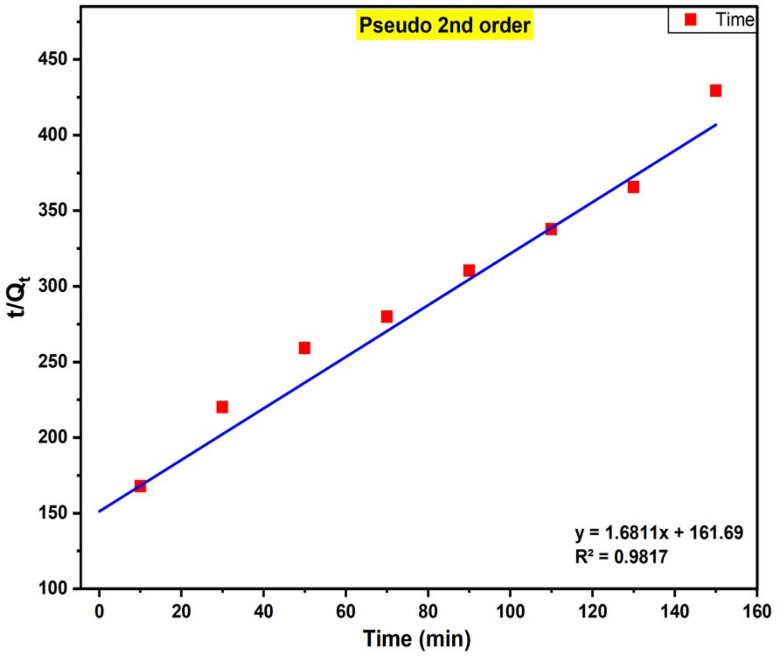

Figure S19b: The plot showing kinetics of Pseudo-second-order for removal of Crystal violet dye by PPy-SnO_2 NCs.

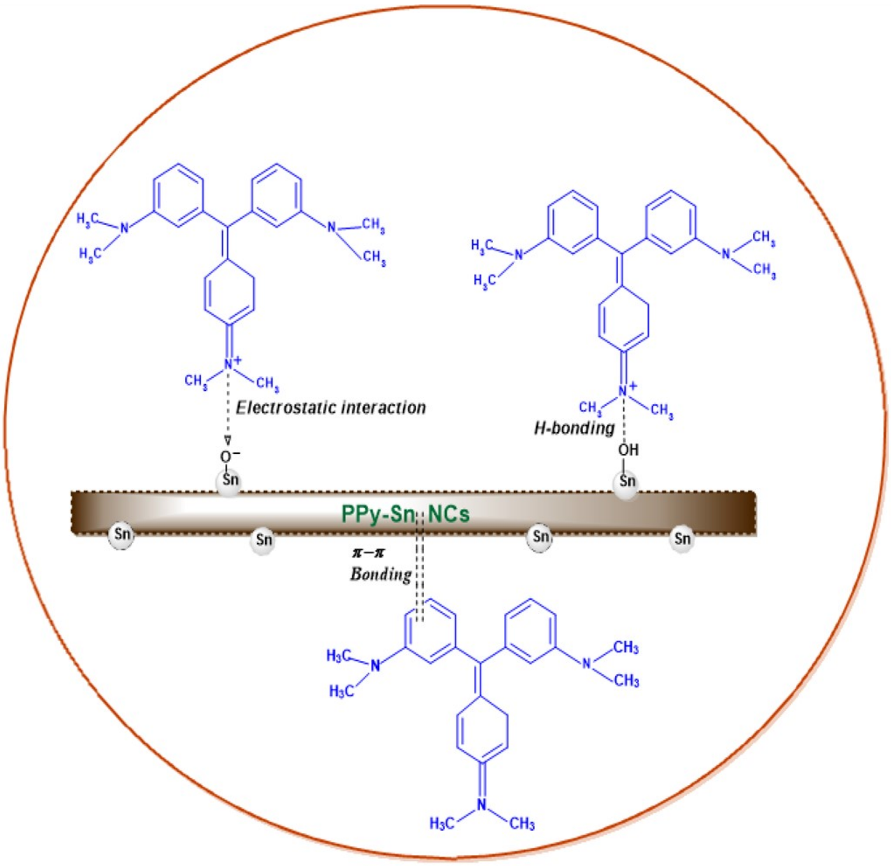

Figure S20: Adsorption of Crystal Violet on PPy–SnO_2 nanocomposites via electrostatic, hydrogen-bonding, and π–π interactions

Table S1: SEM EDS analysis of the synthesized PPy- SnO_2 nanocomposites.

Element	Line	Mass%	Atom%
**C**	K	14.1 ± 0.18	26.6 ± 0.34
**N**	K	3.8 ± 0.30	5.4 ± 0.41
**O**	K	36.3 ± 0.83	57.8± 1.17
**Sn**	L	45.8 ± 1.44	10.2. ± 0.27
**Total**		100.00	100.00

Element	Line	Mass%	Atom%
**C**	K	13.5± 0.2	44.9± 0.3
**N**	K	2.5± 0.19	6.8± 0.2
**O**	K	29.5± 0.8	29.1± 0.9
**Sn**	K	54.5±1.2	19.2± 0.3
**Total**		100	100

Table S2: HRTEM-EDS analysis of the synthesized PPY- SnO_2 nanocomposites.

Table S3: Antioxidant Activity (% DPPH Scavenging)

Sample	Solvent	10 mg (%)	20 mg (%)	30 mg (%)	40 mg (%)
**Ascorbic Acid**	Hexane	23.57± 0.07	28.32± 0.03	35.83 ± 0.11	40.09 ± 0.03
	Methanol	92.67 ± 0.09	97.53 ± 0.11	97.62 ± 0.08	98.21 ± 0.13
**PPy**	Hexane	36.25 ± 0.08	42.57 ± 0.11	52.21 ± 0.05	54.18 ± 0.09
	Methanol	52.90 ± 0.02	55.62 ± 0.03	61.33 ± 0.07	62.95 ± 0.03
**PPy–SnO_2 NCs**	Hexane	45.14 ± 0.13	50.64 ± 0.15	57.92 ± 0.09	58.98 ± 0.11
	Methanol	68.13 ± 0.03	75.92 ± 0.05	83.58 ± 0.07	87.91 ± 0.06

Table S4: Antioxidant Activity (% ABTS Scavenging)

Sample	Solvent	10 mg (%)	20 mg (%)	30 mg (%)	40 mg (%)
**Ascorbic Acid**	Methanol	98.47 ± 0.09	96.83 ± 0.07	99.23 ± 0.03	99.85 ± 0.08
	Hexane	47.87 ± 0.02	53.73 ± 0.05	56.73 ± 0.01	57.21 ± 0.03
**PPy**	Methanol	47.76 ± 0.03	48.78 ± 0.01	53.13 ± 0.03	61.62 ± 0.05
	Hexane	18.16 ± 0.01	25.29 ± 0.03	39.38 ± 0.02	41.51 ± 0.02
**PPy–SnO_2 NCs**	Methanol	65.86 ± 0.03	73.98 ± 0.05	82.76 ± 0.03	90.8 ± 0.02
	Hexane	42.65 ± 0.03	45.28 ± 0.01	51.87 ± 0.03	60.24 ± 0.01

Table S5: Comparison of isotherm and kinetic parameters for crystal violet dye adsorption on PPy–SnO_2 nanocomposites.

Values	Langmuir	Freundlich	pseudo 1st	pseudo 2nd
Q_{max}	162.6	_	_	_
R_2	0.9698	. 0.8946	0.9443	0.998
K_L	0.48	_	_	_
K_f	_	60.9	_	_
n	_	3.09	_	_
Q_e	_	_	0.591	0.598
k_1	_	_	0.221	_
k_2	_	_	_	0.0175

The adsorption performance of the dye on PPy-SnO_2 NCs was evaluated using both isotherm and kinetic models. The Langmuir isotherm suggested monolayer adsorption with a maximum capacity of 162.6 mg·g^{-1} (K_L = 0.48 L·mg^{-1}, R^2 = 0.9698), while the Freundlich model indicated multilayer adsorption on heterogeneous sites (K_F = 60.9, n = 3.09, R^2 = 0.8946). Kinetic analysis showed that the pseudo-first-order model reasonably described the adsorption process ($Q_{e,\ cal}$ = 0.591 mg·g^{-1}, k_1 = 0.221 min^{-1}, R^2 = 0.9443), whereas the pseudo-second-order model provided a slightly better fit ($Q_{e,\ cal}$ = 0.598 mg·g^{-1}, k_2 = 0.0175 g·mg^{-1}·min^{-1}, R^2 = 0.998). Overall, these findings confirm that adsorption is primarily governed by physisorption, highlighting the efficiency of PPy- SnO_2 NCs nanoparticles for dye removal applications.

Table S6: Comparison table for CV dye adsorption performance of PPy-SnO_2 NCs with reported adsorbents.

Adsorbent	qmax (mg g^{-1})	Optimum pH	Temperature (°C)	Contact Time (min)	Reference
**PPy-SnO_2**	162.6	7	50	150	[our study]
**Ferrite-biochar composite**	325.4	7-8	30	120	[64]
**Modified carbon spheres**	134.6	6	25	180	[65]
**Carbon-sphere / Titania-nanotube composite (TNTs@Cs)**	84.7	5.5	25–30	180	[66]
**Natural clay**	203.6	7	25	120	[67]
